# A long noncoding RNA-based serum signature predicts ado-trastuzumab emtansine (T-DM1) treatment benefit in HER2+ metastatic breast cancer patients: a multicenter cohort study

**DOI:** 10.1038/s41420-025-02701-8

**Published:** 2025-09-09

**Authors:** Syed S. Islam, Taher Al-Tweigeri, Asma Tulbah, Saleh N. Najjar, Sarah S. Aljohani, Layla Al-Harbi, Ahmed M. Gad, Shafat Ujjahan, Abdelilah Aboussekhra

**Affiliations:** 1 Department of Molecular Oncology, King Faisal Specialist Hospital & Research Centre, Riyadh, Saudi Arabia; 2 School of Medicine, Al-Faisal University, Riyadh, Saudi Arabia; 3 Department of Breast Oncology, Cancer Centre of Excellence, King Faisal Specialist Hospital & Research Centre, Riyadh, Saudi Arabia; 4 Department of Pathology and Laboratory Medicine, King Faisal Specialist Hospital & Research Centre, Riyadh, Saudi Arabia; 5 Department of Infection and Immunity, King Faisal Specialist Hospital and Research Centre, Riyadh, Saudi Arabia; 6 Department of Radiation Oncology, Chattogram Maa-O-Sishu Hospital, and Park View Hospital, Chattogram, Bangladesh

**Keywords:** Cancer genomics, Prognostic markers

## Abstract

Ado-trastuzumab is considered a standard treatment for patients with HER2+ metastatic breast cancer (mBC). Current clinical practices do not reliably predict therapeutic outcomes for patients who are refractory to therapy. Long noncoding RNAs (lncRNAs) are emerging as critical regulators of gene expression and therapeutic resistance, and the use of lncRNAs as tumor biomarkers is becoming more common in other diseases. However, whether they may also be used to predict therapy response in HER2+ mBC is unclear. Using lncRNA microarray profiling, we identified 23 differentially expressed lncRNAs in the serum of HER2+ mBC patients with unique responses to trastuzumab-emtansine (T-DM1). Following RT-PCR validation and machine learning-based selection in the training cohort, four lncRNAs were selected to construct the signature panel and used for T-DM1 response prediction. This four-lncRNA signature classifies patients into high- and low-risk groups and significantly and distinctively predicts patient survival. Importantly, identical outcomes were obtained from the two validation cohorts, confirming that the signature accurately predicts the T-DM1 response of HER2+ mBC patients. Integrative analysis demonstrated that this four-lncRNA signature is primarily released by immune and tumor cells and is correlated with immune activity. Our findings indicate that the four-lncRNA signature is a potentially promising biomarker for predicting T-DM1 treatment outcome, as it may reliably predict the T-DM1 treatment response in HER2+ mBC.

## Introduction

The overexpression of HER2 in breast cancer (BC) represents 20% of all BC cases and is associated with poor clinical outcomes. Treatment with trastuzumab emtansine (T-DM1) significantly improved the clinical outcomes of patients with HER2+ metastatic breast cancer (mBC). Nevertheless, many patients are refractory to T-DM1 [1, 2]. Therefore, identifying patients who will benefit from T-DM1 is indispensable. In the clinical setting, several tissue biomarkers, including HER2, immune cell signatures, and estrogen receptor (ER) status, have been used to predict chemotherapy sensitivity [3, 4]. In addition, these biomarkers have been used to monitor disease progression and patient survival and determine the optimal course of treatment [5]. Access to tumor tissues is a typical prerequisite for biomarker studies. There are occasions when tumor samples are unavailable for precise biomarker analysis. Nonetheless, studies of biomarkers from biopsy samples are often limited due to intratumoral heterogeneity [6]. Although several tissue predictive markers have been studied in patients with HER2+ mBC who are treated with T-DM1, no study has successfully predicted T-DM1 sensitivity via blood serum.

Long noncoding RNAs (lncRNAs) have garnered significant attention for their diverse regulatory roles in biological and molecular processes owing to the development of deeper and more sensitive RNA sequencing and computational prediction techniques [7, 8]. They are also known to exquisitely govern cell type-specific functions more precisely than regular mRNAs or to regulate discrete genomic regions [9]. LncRNAs in tumor tissues can be used as biomarkers to predict prognostic signatures and treatment outcomes. Additionally, it has been shown that lncRNA signature profiles can accurately predict metastasis [10]. Moreover, recent evidence suggests that blood-derived lncRNAs may contribute to the early detection of malignancies as well as the prognosis for cancer patients’ survival following tumor removal [11–13]. However, few studies have investigated whether serum lncRNA signatures may be used as T-DM1 treatment predictors in HER2 + BC patients.

In this multicenter cohort study, we identified and reported a four-lncRNA signature as a predictor of T-DM1 sensitivity in HER2+ mBC patients. T-DM1 treatment outcome-related lncRNAs were accurately identified by profiling lncRNA expression via microarrays in the training cohort via LASSO Cox regression and machine learning approaches. Two independent cohorts further demonstrated that the signature can accurately predict which patients are more likely to be resistant or susceptible to treatment. Additionally, bioinformatics analysis was employed to explore the source and immune-related characteristics of the four-lncRNA signature. In conclusion, the four-lncRNA signature offers practical T-DM1 treatment-related biomarkers for personalized therapy.

## Results

### Clinicopathological features of patients in the training, internal testing, and independent validation cohorts

**Fig. 1:**
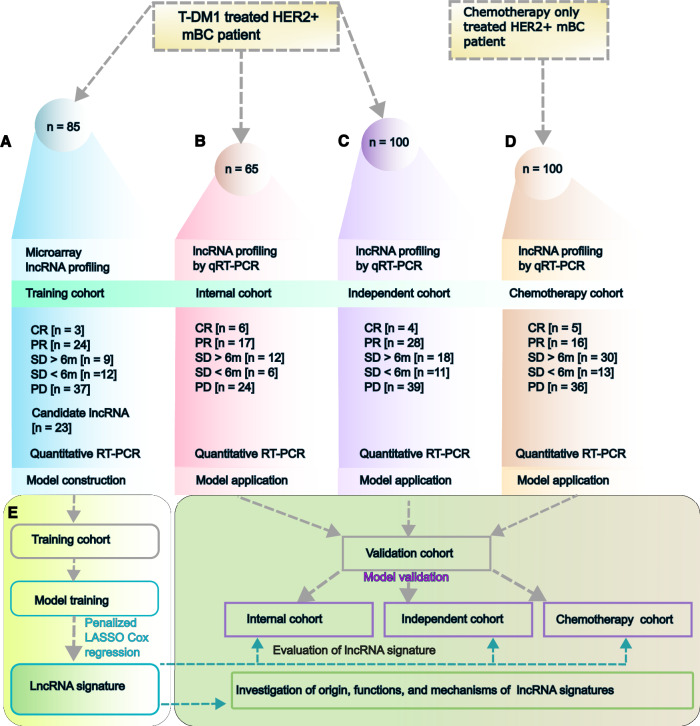
Schematic diagram and study design of HER2 + mBC patients. The datasets are primary samples that were obtained at the time of diagnosis and are classified as follows: **A** training cohort (blue), **B** internal cohort (red), **C** independent cohort (purple), and **D** chemotherapy-only cohort (orange-red). Microarray data generated from (**A**). **E** Study design for the training cohort and two validation cohorts (internal and independent cohorts) for lncRNA signature discovery and functional analysis of the lncRNA signature. Patients who achieved CR, PR, or SD for more than 6 months were defined as sensitive to T-DM1. PD or SD prolonged to less than 6 months was defined as resistance to T-DM1. CR complete response, PR partial response, SD stable disease, PD progressive disease.

**Fig. 2:**
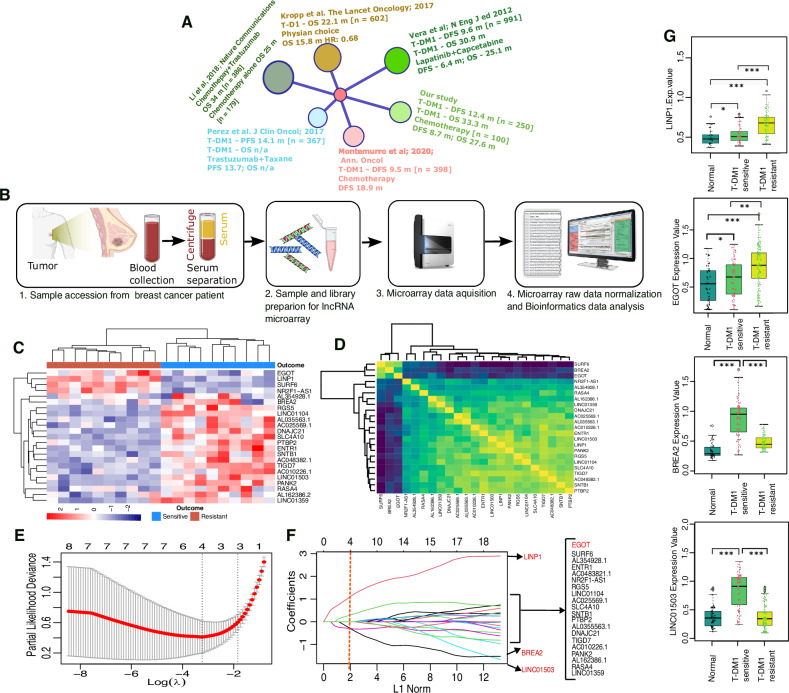
Development of the lncRNA-based signature. **A** Network plot showing the comparisons of overall survival (OS) and disease-free survival (DFS) between our study and several previously published studies. **B** Schematic diagram of the workflow for developing a serum-based signature. **C** Hierarchical clustering and heatmap of 23 differentially expressed lncRNAs from 10 HER2+ mBC patient serum samples resistant (red) to T-DM1 treatment and 10 HER2+ mBC patient serum samples responsive (deep blue) to T-DM1 treatment. Euclidean distance and average linkage clustering were used. Each row represents an individual lncRNA, and each column represents an individual sample. The pseudocolor indicates the lncRNA level from high to low. **D** Hierarchical clustering correlation plot showing the collinearity of 23 candidate lncRNAs. Correlation matrix heatmap of 23 lncRNAs in the training cohort, where each cell represents the Pearson correlation between the row and column corresponding lncRNAs. The legend shows the color change along with the changes in the correlation coefficient from −1.0 to 1.0. **E** Four lncRNAs selected by LASSO Cox regression analysis. Two hundred cross-validations for tuning parameter selection in the LASSO model corresponding to Fig. 1D. The two dotted vertical lines mark the optimal values according to the minimum criteria and the 1-s.e. criteria. **F** LASSO coefficient profiles of the 23 HER2+ mBC-associated serum lncRNAs. The vertical red dotted line indicates the optimal value based on the 1-s criterion, which gives four nonzero coefficients: LINP1, 0.575; EGOT, 0.29; BREA2, −0.64; and LINC01503, −0.42. **G** qRT-PCR analysis of the expression levels of the 4 lncRNAs in healthy individuals and T-DM1-resistant and T-DM1-sensitive patients. The error bars in the graphs represent the standard deviation (s.d.). *p < 0.05, **p < 0.01, ***p < 0.001. *p* values were obtained via two-tailed Student’s *t* tests.

This investigation included 250 (*n* = 250) patients with HER2+ mBC who were receiving trastuzumab emtansine (T-DM1) treatment. The study design of each cohort is shown in Fig. 1A–E and Supplementary Fig. S1. The patient characteristics of the training cohort (*n* = 85), internal cohort (*n* = 65), independent cohort (*n* = 100), and chemotherapy-only cohort (*n* = 100) are shown in Table 1 and Supplementary Table S2. The age, treatment history, and molecular subtypes of each cohort were uniform and well balanced. The median follow-up durations were 36 months (interquartile range: IQR 20–39), 40.2 months (IQR 19–45), 32.1 months (IQR 18–39), and 40.2 months (IQR 15–43) in the training, internal, independent, and chemotherapy-only cohorts, respectively. We searched for the treatment outcomes of HER2+ mBC patients published previously and found that our results are comparable to those that have already been published (Fig. 2A).

**Table 1:** Baseline clinical characteristics of patients from 250 HER2 + mBC patients receiving T-DM1 treatment by lncRNA assessment set.

Patients characteristics	Chemotherapy cohort			T-DM1 treatment cohort								
				Training cohort			Internal cohort			Independent cohort		
	High score	Low score	*p* value^a^	High score [*n* = 45]	Low score [*n* = 41]	*p* value^a^	High score [*n* = 34]	Low score [*n* = 31]	*p* value^a^	High score [*n* = 44]	Low score [*n* = 56]	*p* value^a^
Age, median, years, range	53 [37–71]	55 [37–72]	0.2	48 [44–62]	47 [45–66]	0.7	53 [47–64]	50 [42–61]	0.14	55 [48–58]	55 [49–62]	0.8
Menopause status, *n*, [%]												
Pre-menopausal	15 (36)	12 (21)	0.095	26 (58)	22 (54)	0.7	16 (47)	15 (48)	0.91	13 (30)	15 (27)	0.8
Post-menopausal	27 (64)	46 (79)		19 (42)	19 (46)		18 (53)	16 (52)		31 (70)	41 (73)	
Ki67 rate												
<20%	5 (12)	8 (14)	0.9	5 (11)	8 (20)	0.6	10 (29)	8 (26)	0.5	4 (9.1)	5 (8.9)	0.95
>20%	33 (79)	46 (79)		38 (84)	31 (76)		19 (56)	21 (68)		36 (82)	44 (79)	
Unidentified	4 (9.5)	4 (6.9)		2 (4.4)	2 (4.9)		5 (15)	2 (6.5)		4 (9.1)	7 (12)	
ECOG performance status, *n*, [%]												
0	30 (71)	33 (57)	0.3	31 (69)	24 (59)	0.6	25 (74)	18 (58)	0.2	33 (75)	31 (55)	0.062
1	8 (19)	19 (33)		12 (27)	15 (37)		8 (24)	8 (26)		6 (14)	19 (34)	
2	4 (9.5)	6 (10)		2 (4.4)	2 (4.9)		1 (2.9)	5 (16)		5 (11)	6 (11)	
Stage, *n*, [%]												
I	8 (19)	10 (17)	>0.9	9 (20)	7 (17)	0.4	8 (24)	6 (19)	0.93	9 (20)	14 (25)	0.91
II	7 (17)	8 (14)		19 (42)	12 (29)		13 (38)	14 (45)		21 (48)	27 (48)	
III	19 (45)	27 (47)		13 (29)	14 (34)		8 (24)	7 (23)		9 (20)	10 (18)	
IV	8 (19)	13 (22)		4 (8.9)	8 (20)		5 (15)	4 (13)		5 (11)	5 (8.9)	
ER- status, *n*, [%]												
Positive	20 (48)	24 (410	0.5	28 (62)	27 (66)	0.7	24 (71)	19 (61)	0.4	25 (57)	32 (57)	0.96
Negative	22 (52)	34 (59)		17 (38)	14 (34)		10 (29)	12 (39)		19 (43)	24 (43)	
PR- status, *n*, [%]												
Positive	19 (45)	27 (47)	0.9	28 (62)	27 (66)	0.7	18 (53)	13 (42)	0.4	21 (48)	30 (54)	0.6
Negative	23 (55)	31 (53)		17 (38)	14 (34)		16 (47)	18 (58)		23 (52)	26 (46)	
Number of metastatic sites, *n*, [%]												
1	18 (43)	30 (52)	0.4	17 (38)	8 (20)	0.4	15 (44)	14 (45)	0.6	22 (50)	26 (46)	0.9
2	16 (38)	15 (26)		16 (36)	23 (56)		12 (35)	8 (26)		12 (27)	15 (27)	
3 or more	8 (19)	13 (22)		12 (27)	10 (24)		7 (21)	9 (29)		10 (23)	15 (27)	
Metastatic sites, *n*, [%]												
Bone	7 (17)	9 (16)	0.4	18 (40)	11 (27)	0.5	12 (35)	10 (32)	0.8	11 (25)	16 (29)	0.9
Soft tissues	16 (38)	26(45)		6 (13)	6 (15)		4 (12)	6 (19)		8 (18)	11 (20)	
Visceral	15 (36)	22 (38)		13 (29)	9 (22)		8 (24)	9 (29)		14 (32)	16 (29)	
Brain	4 (9.5)	1 (1.7)		3 (6.7)	4 (9.8)		4 (12)	4 (13)		6 (14)	6 (11)	
Lung	n/a	n/a		3 (6.7)	6 (15)		3 (8.8)	1 (3.2)		4 (9.1)	3 (5.4)	
Liver	n/a	n/a		2 (4.4)	5 (15)		3 (8.8)	1 (3.2)		1 (2.3)	4 (7.1)	
Chemotherapy, *n*, [%]												
Paclitaxel	8 (19)	10 (17)	0.9	11 (24)	9 (22)	0.4	8 (24)	3 (9.7)	0.3	9 (20)	10 (18)	0.4
Docetaxel	20 (48)	24 (41)		21 (47)	13 (32)		11 (32)	15 (48)		18 (41)	25 (45)	
Doxorubicin	10 (24)	17 (29)		9 (20)	12 (29)		10 (29)	7 (23)		14 (32(	12 (21)	
Capecitabine	4 (9.5)	7 (17)		4 (8.9)	7 (17)		5 (15)	6 (19)		3 (6.8)	9 (16)	
Response to treatment, *n*, [%]												
CR	1 (2.4)	4 (6.9)	0.037	1 (2.3)	2 (4.9)	0.020	1 (2.9)	5 (13)	0.015	1 (2.3)	3 (5.4)	0.038
PR	2 (4.8)	14 (24)		6 (13)	18 (44)		6 (18)	11 (35)		7 (16)	21 (38)	
SD > 6 months	14 (33)	16 (28)		5 (11)	4 (9.8)		4 (12)	8 (26)		7 (16)	11 (20)	
SD < 6 months	5 (12)	8 (14)		7 (16)	5 (12)		5 (15)	1 (3.2)		5 (11)	6 (11)	
PD	20 (48)	16 (28)		25 (56)	12 (29)		17 (53)	7 (23)		24 (55)	15 (27)	

*CR* complete response, *SD* stable disease, *PR* partial response, *PD* progressive disease, *ER* estrogen receptor, *PR* progesterone receptor, *ECOG* Eastern Cooperative Oncology Group.

^a^Fisher’s exact test or Chi-square test.

### Microarray profiling and development of a serum lncRNA signature for the response to T-DM1 treatment

To precisely ascertain the serum lncRNA signature, we obtained data from a group of 85 patients referred to as the training cohort. In the training cohort, 49 patients were resistant to T-DM1 treatment, whereas 36 patients responded to T-DM1. Using a random selection process, 20 of the 85 patients from both the resistant and sensitive patient groups were selected for microarray analysis, in which we looked for differentially expressed lncRNAs. Figure 2B shows a schematic diagram of the working flowchart for sample collection, sample preparation, and microarray data acquisition. On the basis of the lncRNA microarray data, we identified 23 lncRNAs differentially expressed across sensitive and resistant samples (*p* < 0.05) among all the lncRNA candidates. In the sensitive group, a total of 4 lncRNAs were downregulated (LINP1, EGOT, SURF6, and NRF2F1-AS1), and 19 lncRNAs (AL354928.1, BREA2, RGS5, LINC01104, AL0355563.1, AC025569.1, DNAJC21, SLC4A10, PTBP2, ENTR1, SNTB1, AC048382.1, TIGD7, AC010226.1, LINC01503, PANK2, RASA4, AL162386.1, and LINC01359) were upregulated (Fig. 2C).

We utilized quantitative reverse transcriptase‒PCR (qRT‒PCR) to analyze the expression of 23 differentially expressed lncRNAs from the training cohort to construct a straightforward yet effective model. To reduce the number of potential lncRNAs, we applied the machine learning method. Univariate Cox regression analysis revealed that several lncRNAs were collinear (Fig. 2D). This led us to construct an lncRNA signature via the least absolute shrinkage and selection operator (LASSO) method with 200 bootstrap replicates via penalized maximum likelihood for the prediction of progression-free survival (PFS) in the training cohort (Fig. 2E, F). For LASSO, the regularization path was computed at a grid of values for the lambda (λ) regularization parameters. This analysis revealed 4 lncRNAs (LINP1, EGOT, BREA2, and LINC01503) among the 23 lncRNAs (Fig. 2E, F). To construct a prognostic signature, we generated a risk score for every patient via a formula that included the 4 selected lncRNAs weighted by their regression coefficients in the penalized Cox model. The risk score was calculated as follows: (0.574 × expression value of lncRNA-LINP1) + (0.29 × expression value of lncRNA EGOT) + (−0.42 × expression value of lncRNA-BREA2) + (−0.64 × expression value of lncRNA LINC01503).

To determine how these 4 lncRNAs are expressed in normal females, we randomly collected serum samples from 50 healthy donors. The findings revealed no appreciable differences in the serum levels of BREA2 and LINC01305 between HER2+ mBC patients who were sensitive to T-DM1 treatment and normal donors. Nevertheless, the levels of these two lncRNAs decreased in HER2+ mBC patients who were not responsive to T-DM1 treatment. In contrast, the levels of the lncRNAs LINP1 and EGOT were greater in HER2+ mBC patients who did not respond to T-DM1 treatment than in normal donors, and they were even greater in patients who were sensitive to T-DM1 treatment (Fig. 2G). To confirm the correlation between the serum and tissue lncRNA expression levels, the expression levels of 4 selected lncRNAs from a small set (*n* = 15) from the training dataset were analyzed. The lncRNA LINP1 and EGOT expression levels were significantly higher in resistant patients than in sensitive patients (Supplementary Fig. S2A). The tissue lncRNA results corroborated the serum lncRNA levels, confirming a statistically significant correlation between resistant and sensitive tissues and matched serum lncRNAs (Supplementary Fig. S2B).

### Evaluation of the optimal cutoff value and generation of a risk score for signature lncRNAs

**Fig. 3:**
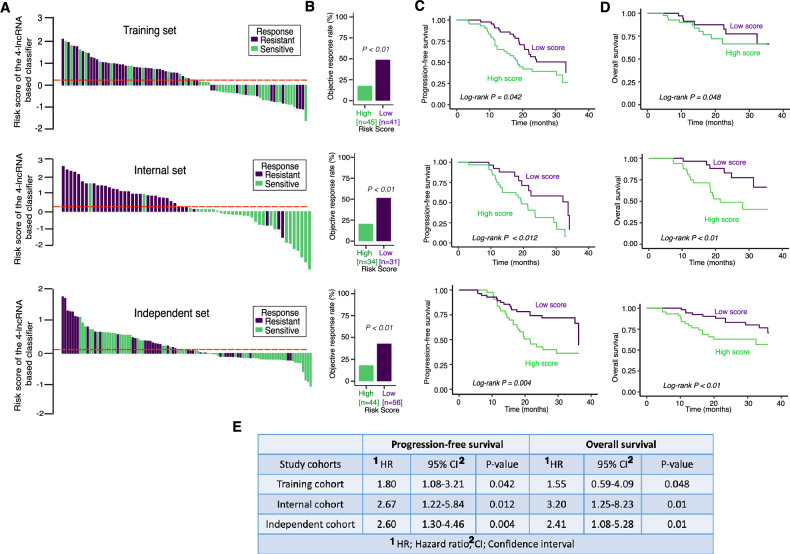
Distribution of the risk score, T-DM1 response, overall survival (OS), and progression-free survival (PFS) based on the 4-lncRNA classifier in the training, internal, and independent validation cohorts. **A** Waterfall plot for the distribution of the risk score and response status of individual patients in the training (top panel), internal (middle panel), and independent (bottom panel) cohorts. **B** Objective response rates of the high- and low-score groups in the training (top panel), internal (middle panel), and independent (bottom panel) cohorts. **C** Comparison of PFS between the high- and low-score groups in the training (top panel), internal (middle panel), and independent (bottom panel) cohorts. **D** Comparison of OS between the high- and low-score groups in the training (top panel), internal (middle panel), and independent (bottom panel) cohorts. **E** Hazard ratios and *p* values for PFS and OS in the training, internal, and independent cohorts. We calculated hazard ratios (HRs) and *p* values via univariate Cox regression analysis and the log-rank test. All the statistical analyses were two-sided.

Patients in the training cohort were divided into two groups on the basis of the optimum cutoff score (Fig. 3A, top panel; Supplementary Fig. S3). Patients with a risk score greater than 0.17 were classified as having a high risk of PFS, whereas those with a risk score less than 0.17 were classified as having a low risk of PFS. With this optimal cutoff value, 45 patients in this training cohort were classified into the high-score group, and the remaining 41 patients were classified into the low-score group (Table 1). There was no substantial difference (*p* < 0.05) in the overall pattern of clinicopathological features between the high-risk and low-risk groups (Table 1). Nonetheless, when considering the treatment response across the two groups, we found that patients with low scores responded better to T-DM1 treatment than did those in the high-risk group (Fig. 3B; top panel). In addition, the objective response rate (ORR) was 18.8% (8/45) for the high-risk group (Fig. 3B; top panel) and 48.78% (20/41) for the low-risk group (*p* < 0.01; Fig. 3B; top panel). Furthermore, patients in the high-score group exhibited worse PFS and OS (Fig. 3C, D; top panel). The PFS was 18.2 vs. 26.2 months (HR 1.80, 95% CI 10.01–3.21; *p* = 0.042), whereas the OS was 22.1 vs. 33.0 months (HR 1.55, 95% CI 0.59–4.09; *p* = 0.048).

### Validation of signatures in internal and external independent cohorts

To corroborate the value and efficacy of the 4-lncRNA signature in predicting PFS and OS, we validated our findings in two additional internal and independent cohorts. We applied identical algorithms and cutoff points that were established in the training cohort. Of the 65 patients in the internal cohort, 34 were classified as high risk, and 31 were classified as low risk. An analysis of the two groups’ responses to T-DM1 treatment and ORR revealed substantial disparities. As shown in Fig. 3B (middle panel), in the internal cohort, the low-risk group had a better response to T-DM1 treatment than the high-risk group did, with the ORR rates for the low-risk group being 51.61% (16/31) and that for the high-risk group being 20.58% (7/34) (*p* < 0.01). The median PFS was 20.2 vs. 32.0 months (Fig. 3C, E; middle panel; HR 2.67, 95% CI 1.22–5.84, *p* = 0.012), and the median OS was 21.6 vs. >3 years (Fig. 3B, E; middle panel; HR 3.20, 95% CI 1.25–8.23, *p* < 0.01). Following a similar method and cutoff setting applied to the training and internal cohorts, 44 of the 100 patients in the independent cohort were classified as high risk, whereas the remaining 56 patients were classified as low risk. Patients in the high-risk group responded the least to T-DM1 treatment, as seen in the internal and training cohorts (Fig. 3B; bottom panel). The ORR was 18.18% (8/44) for the low-risk group and 42.84% (24/55) for the low-risk group (Fig. 3B, bottom panel; *p* < 0.01). As expected, the high-risk group had shorter PFS and OS than the low-risk group did (PFS: HR 2.60, 95% CI 1.30–4.46, *p* < 0.004; OS: HR 2.41, 95% CI 1.08–5.28, *p* = 0.01; Fig. 3C–E; bottom panel).

### Univariate and multivariate analyses of the training, internal, and independent validation cohorts

**Fig. 4:**
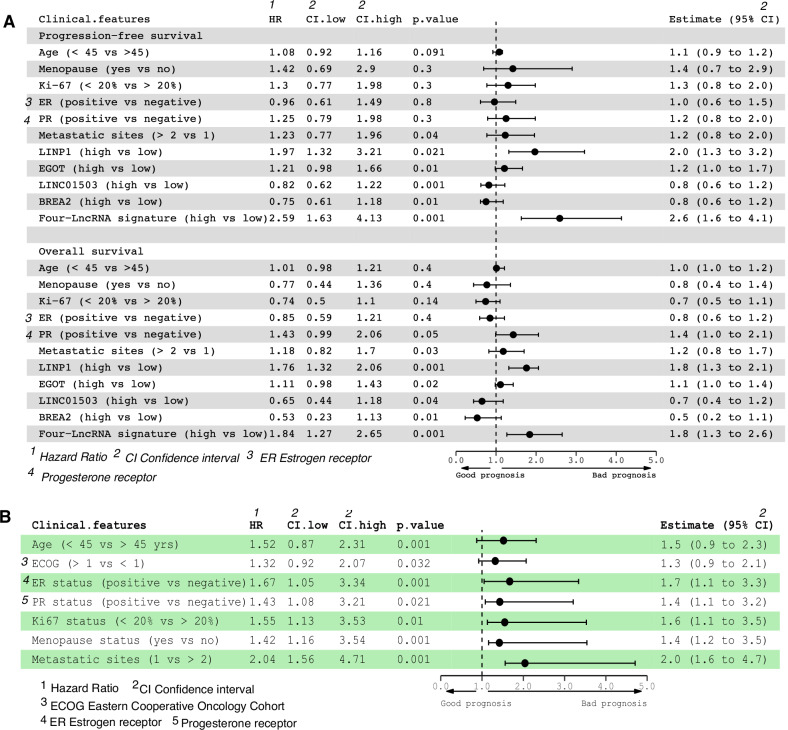
The four-lncRNA classifier identified suitable candidates for PFS and OS in different patient subgroups. **A** Forest plot depicting multivariate Cox regression model analysis of clinical features and survival status in the entire cohort (training, internal, and independent validation cohorts). *p* values were calculated via the two-sided log-rank test. **B** Forest plot for the performance of the Cox model in predicting the risk score in terms of PFS stratified by clinicopathological features. *p* values were calculated via the two-sided log-rank test. HRs and 95% CIs are given and are visually represented by circles and error bars. ECOG Eastern Cooperative Oncology Cohort, ER estrogen receptor, PR progesterone receptor, HR hazard ratio, CI 95% confidence interval.

Univariate and multivariate analyses of clinical variables for PFS and OS were carried out in three cohorts. Univariate analysis revealed that each lncRNA was an independent prognostic factor for T-DM1 treatment (Supplementary Table S3). For PFS and OS, multivariate survival analysis was performed using the 4-lncRNA signature and associated clinical and pathological characteristics. The 4-lncRNA signature and metastatic sites were found to be distinct prognostic variables (Fig. 4A). After stratification and adjustment for age, ECOG score, ER status, PR status, Ki-67 index, menopause status, and metastatic site, the 4-lncRNA signature remained a powerful, independent, and significant predictor of PFS (Fig. 4B).

### The 4-lncRNA signature outperforms traditional clinicopathological factors

**Fig. 5:**
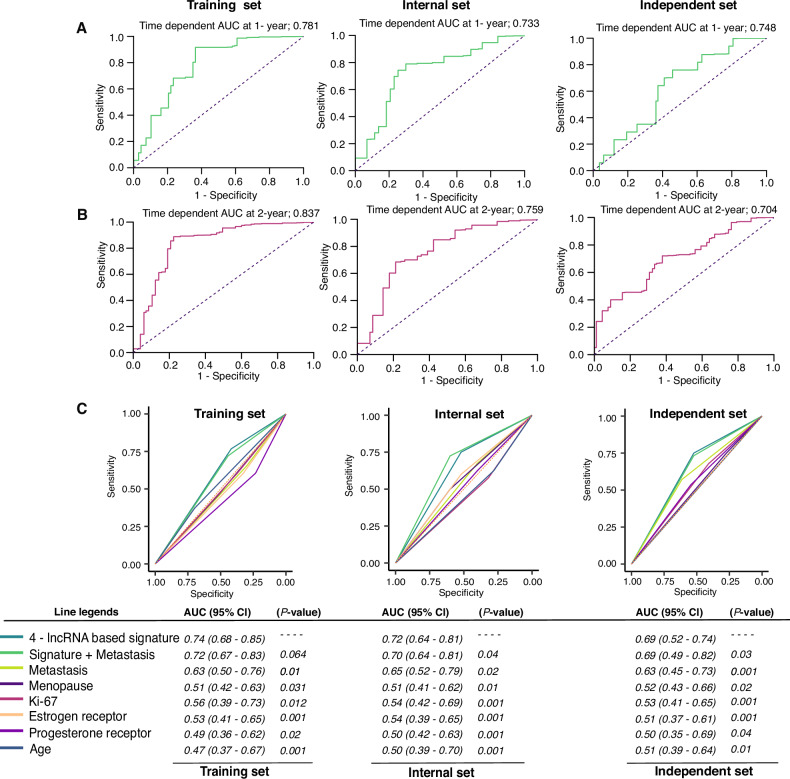
Time-dependent receiver operating characteristic (ROC) curves and areas under the curve (AUCs) in the training, internal, and independent validation cohorts. **A**, **B** The time-dependent area under the ROC curve (AUC) at 1 and 2 years was used to assess the prognostic accuracy of the 4-lncRNA signature. **C** Comparison of the prognostic accuracy of the 4-lncRNA signature (high vs. low score) for T-DM1 response in patients with different clinicopathological characteristics. *p* values represent the AUC of the 4-lncRNA signature vs. the AUC of the clinical features. The AUC and 95% CI were calculated via the bootstrap method. The *p* values were two-sided on the basis of the bootstrap test.

Metastasis, other clinicopathological risk factors, or lncRNAs alone were associated with OS and PFS according to univariate Cox analysis (Fig. 4A, B). We then evaluated the predictive accuracy of the 4-lncRNA signature with these variables. To demonstrate the predictive accuracy, we computed the area under the receiver operating characteristic (AUC) curve (AUC) for the training, internal, and independent cohorts. The 4-lncRNA signature demonstrated superior efficacy compared with other clinicopathological parameters according to the results of the time-dependent receiver operating characteristic (ROC) analysis (Fig. 5A–C). The AUCs for 1- and 2-year OS with the 4-lncRNA signature were 0.781 and 0.837, respectively, for the training cohort (Fig. 5A, B); 0.733 and 0.759, respectively, for the internal cohort (Fig. 5A, B); and 0.748 and 0.704, respectively, for the independent cohort (Fig. 5A, B). We then analyzed the predictive accuracy of the 4-lncRNA signature with every single lncRNA and found that the 4-lncRNA signature together had a greater AUC (0.74, 95% CI 0.65–0.83), specificity (75.2%), and sensitivity (78.1%) in the training and two validation cohorts (internal cohort: AUC 0.76, 95% CI 0.66–0.87, specificity 74.3%, sensitivity 75.1%; independent cohort: AUC 0.77, 95% CI 0.69–0.85; specificity 78.3%, sensitivity 73.3%). Compared with other clinicopathological risk factors, the signature exhibited superior sensitivity and specificity (*p* < 0.05) in the training and validation cohorts (Fig. 5C).

### The predictive value of the 4-lncRNA signature for the T-DM1 response

**Fig. 6:**
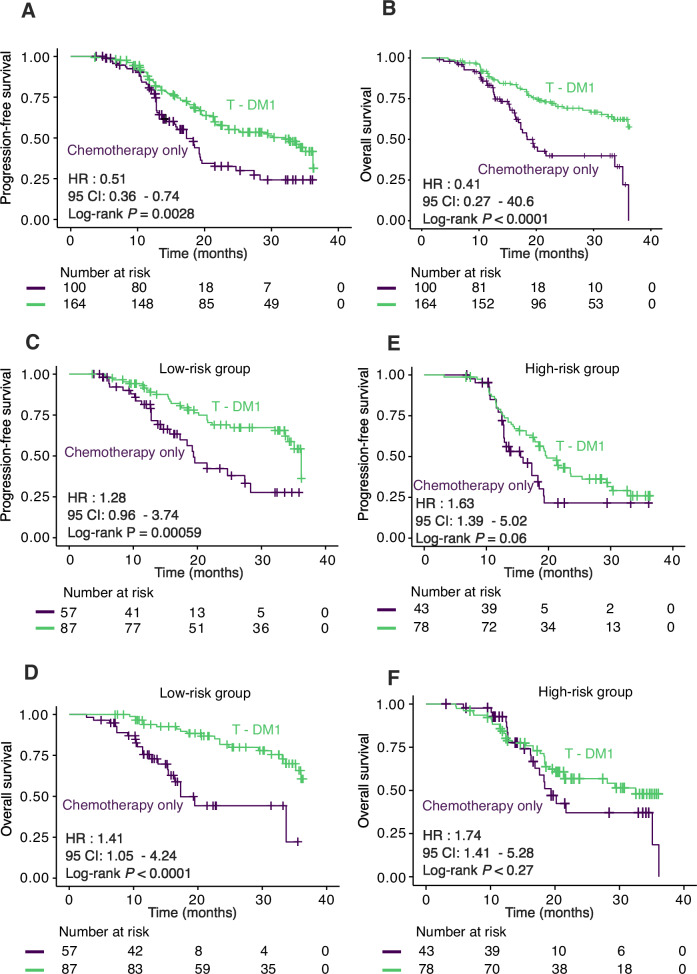
Survival prediction of HER2 + mBC patients by the 4-lncRNA signature in chemotherapy-only patients. **A**, **B** Kaplan‒Meier survival analysis of patients in the chemotherapy cohort and two validation cohorts according to the 4-lncRNA signature and T-DM1 therapy. **C**–**F** The therapeutic advantage was evaluated via Kaplan‒Meier analysis. Patients were segregated according to treatment options into high- and low-score groups. *p* values were calculated with the two-sided log-rank test.

We employed a cohort of patients (*n* = 100) who received only first-line chemotherapy to investigate whether the 4-lncRNA signature represents a distinct predictive marker of T-DM1 sensitivity or merely a prognostic marker for adverse clinical outcomes. Table 1 displays the clinicopathological features of the patients in the chemotherapy-only cohort. We explored whether the 4-lncRNA signature was indeed predictive by contrasting the clinical results of the two validation cohorts (*n* = 165) receiving T-DM1 as a treatment regimen with those of the cohort receiving only chemotherapy. Importantly, there was no significant difference in age or other clinicopathological parameters between the cohorts receiving chemotherapy alone and those receiving T-DM1 in the validation cohort (Supplementary Table S1). Compared with T-DM1 treatment, chemotherapy alone did not improve PFS (HR 0.51; 95% CI 0.36–0.74; *p* = 0.0028) or OS (HR 0.41; 95% CI 0.25–0.63; *p* < 0.0001) (Fig. 6A, B). An ad hoc exploratory subset analysis using our 4-lncRNA-based signature revealed that the low-risk group had a favorable response to T-DM1 and had prolonged PFS (HR 1.28, 95% CI 0.96–3.74; *p* = 0.00059) or OS (HR 1.41; 95% CI 1.05–4.24; *p* < 0.0001) (Fig. 6C, D). In contrast to what was found in Fig. 6C, D, PFS (HR 1.63; 95% CI 1.39–5.02, *p* = 0.06) and OS (HR 1.74; 95% CI 1.41–5.28; *p* = 0.27) were not significantly different between the chemotherapy alone group and the high-risk group in the T-DM1 subgroup (Fig. 6E, F). These findings indicate that our signature/classifier accurately predicts treatment responses/outcomes that are suitable for T-DM1 treatment.

### Biological characteristics, origin, and mechanism of action of the lncRNA signatures

**Fig. 7:**
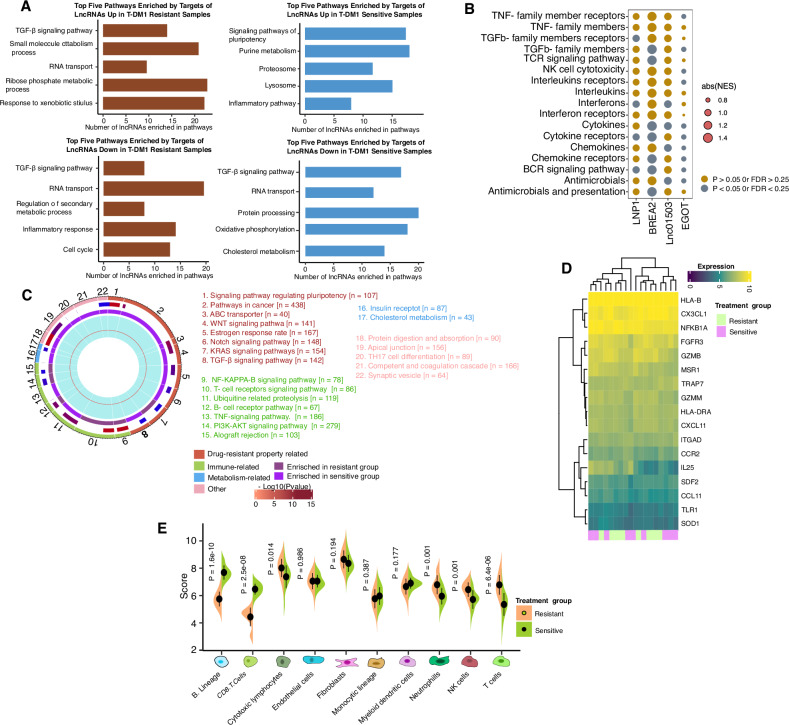
Molecular characteristics of the high-risk and low-risk groups. **A** Bar plot showing the number of lncRNAs out of the four lncRNAs in the signature that are enriched in that corresponding pathway by functional enrichment analysis on the basis of GSEA. The permutation-based *p* value shows the statistical significance of the normalized enrichment score (NES). Adjustments for multiple comparisons are presented by the false discovery rate (FDR). Significantly enriched pathways were defined as those with *p* < 0.05, FDR < 0.25, and absolute NES > 1. **B** Circos plot showing the significantly enriched pathways between the T-DM1 treatment-resistant and T-DM1-sensitive groups based on GSEA. The size of each sector represents the number of genes in the labeled pathway. The color of the outermost area represents the magnitude of the statistical significance. The color of the third circle indicates that the labeled pathways are upregulated in the resistant and sensitive groups. The innermost circle shows the absolute NES. **C** Heatmap of differentially expressed immune-related genes between the T-DM1-resistant and T-DM1-sensitive groups (Student’s *t* test, *p* value < 0.05). FDR false discovery rate, NES normalized enrichment score. **D** Bubble plot of GSEA results of the four lncRNAs with immunologically relevant pathways in HER2-positive mBC patients of the training cohort. The yello-wish golden colour dots represent enrichment (P 0.05, FDR 0.25, absolute NES 1), and the blue colour represents absolute NES. **E** Immune infiltration of patients estimated by the MCP-counter algorithm. The dots represent the mean scores, and the error Bars represent the standard deviations. The comparison was based on Student’s two-sided t-test.

The functional enrichment analysis of the 4 lncRNAs in the signature from the microarray data was performed. Our functional analysis revealed that, in addition to the pathways highly correlated with treatment resistance and tumorigenesis and metastasis, many of the 4-lncRNA signatures were also associated with immune-related pathways (Fig. 7A). To further investigate this finding, we focused on 17 immunologically relevant gene sets obtained from the ImmPort website [14]. The results indicate that the signature lncRNAs are involved in immune pathways, especially those related to T-cell receptor (TCR) signaling and B-cell receptor (BCR) signaling (Fig. 7B). Next, we investigated the associations of the 4-lncRNA signature with immune functions, which we identified among the 4 individual lncRNAs. By comparing the gene expression of the high-risk and low-risk groups on the basis of the lncRNA signature, we found that the genes upregulated in the high-risk group were enriched primarily in malignant property-related and metabolism-related pathways, whereas the genes upregulated in the low-risk group were enriched mainly in immune-related pathways. These findings suggest that the lncRNA signature may be able to distinguish tumors with distinct immune heterogeneity (Fig. 7C). As shown in Fig. 7C, the expression of immune-related genes, resistance pathways, and metastasis pathways differed distinctly between patients in the different treatment groups. Furthermore, the expression of immune-related genes, such as those related to cytotoxic functions, chemokines and cytokines, antigen presentation, and immune cell infiltration, was significantly greater in the resistant and sensitive groups (Fig. 7D). Given that genes representing various immune cell types were differentially expressed between the high- and low-risk groups, we investigated whether the immune cell infiltration pattern also differed between the two groups. The level of immune infiltration was estimated via the MCP-counter algorithm via our microarray data. To distinguish between high- and low-risk patients, we computed the risk score of each patient in the training cohort via qRT-PCR. Patients in the low-risk group had greater levels of B cells, CD8+ T cells, and monocyte infiltration (Fig. 7E).

**Fig. 8:**
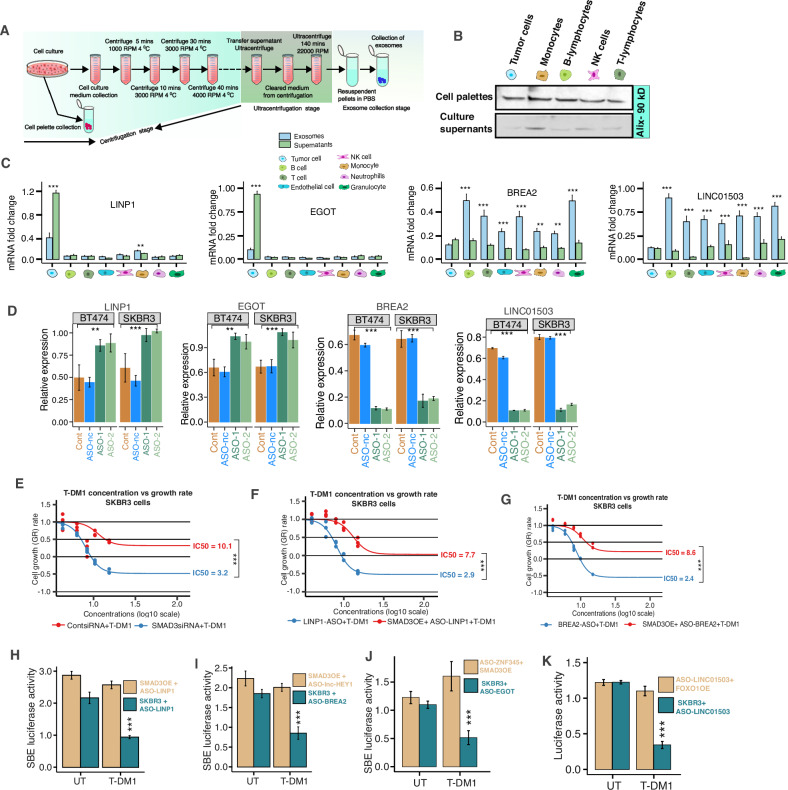
Origin and mechanisms of the lncRNA signature for T-DM1 resistance. **A** Schematic diagram of the culture, collection, and processing of exosomes from supernatants and cultured cells. **B** Western blot analysis of Alix in EVs originating from cell pellets and culture supernatants. **C** Relative expression levels of the 4-lncRNA signature in exosomes and exosome-free supernatants cultured from primary breast tumor cells. Immune cells were isolated from 10 patients (mean + s.e.m. **D** BT474 and SKBR3 cells were transduced with ASO-nc, an ASO of four lncRNAs, and then T-DM1 was added to the culture medium at 8.00 μg/ml for 48 h. The relative expression levels of individual lncRNAs were assessed via qRT-PCR. **E**–**G** A WST-1 assay was used to assess the growth of treated cells as described in (**D**) (mean ± s.e.m., *n* = 3 separate experiments). ***p < 0.001. *p* values were obtained via two-tailed Student’s *t* tests. **H**–**K** Luciferase reporter assay of SKBR3 cells transfected with the indicated vectors in the presence or absence of the indicated lncRNAs. (mean ± s.e.m., *n* = 3 separate experiments). ***p < 0.001. *p* values were obtained via two-tailed Student’s *t* tests.

To validate and explore the origins of the four lncRNAs, we isolated tumor cells from surgically resected samples and isolated autologous T lymphocytes, B lymphocytes, natural killer (NK) cells, monocytes, and granulocytes from the peripheral blood of 10 HER2+ primary breast cancer patients (Fig. 8A). We confirmed the presence of isolated EVs via the EV-specific markers Alix and CD63 (Fig. 8B and Supplementary Fig. S4A). The isolated tumor and immune cells were cultured for 48 h. After 48 h, lncRNAs were extracted from the exosomes and exosome-free culture supernatants in the conditioned medium as described previously [15, 16]. We found that EGOT and LINP1 were highly expressed in exosome-free supernatants derived from tumor cells. On the other hand, BREA2 and LINC01305 were highly expressed in B cells, T cells, NK cells, and granulocytes and moderately expressed in endothelial cells, neutrophils, and monocytes (Fig. 8C). These findings indicate that tumor cells and mixed immune cells are the most likely sources of the 4-lncRNA signature.

Next, how the 4-lncRNA signature contributes to the susceptibility of HER2+ breast cancer cells to T-DM1 was determined. Two distinct ASOs (antisense oligonucleotides) of LINP1, EGOT, BREA2, and LINC01503 were used to transfect SKBR3 and T474 cells, and the cells were then treated with T-DM1 (8 μg ml^−1^) for 48 h (Fig. 8D). Compared with those of cells that were left untreated or treated with ASO-negative control oligonucleotide (Fig. 8D), the ability of LINP1- and EGOT-knockdown cells to invade a Boyden chamber invasion assay was greater, and the ability of these cells to form colonies in Matrigel-coated chambers was abolished (Supplementary Fig. 4B–D). On the other hand, overexpressing LINP1 and EGOT and silencing BREA2 and LINC01503 significantly reduced the sensitivity of tumor cells to T-DM1 and increased their ability to invade and increase their colony formation capacity (Fig. 8D and Supplementary Fig. 4A–D). These findings suggest that the upregulation of LINP1 and EGOT and the downregulation of BREA2 and LINC01305 induce T-DM1 resistance in tumor cells.

To investigate the potential targets and mechanism of the T-DM1 resistance of these lncRNA signatures, we used the target prediction tools LncTar and LncTarD 2.0 [17, 18]. Among the candidate target genes, SMAD3 for LINP1, BREA2, and EGOT and FOXO1 for LINC01305 were potential targets of the signature. It has been reported that aberrant SMAD3 and FOXO1 signaling is involved in trastuzumab resistance in breast cancer cells [19, 20]. To further validate these targets, we first knocked down SMAD3 with a SMAD3-specific siRNA and treated the cells with T-DM1 (8 μg/mL). When endogenous SMAD3 was effectively silenced, the IC_50_ values of SKBR3 cells significantly increased (Fig. 8E). To overexpress SMAD3, we transfected SKBR3 cells with a plasmid containing pc-DNA3.1-SMAD3 and assessed cell growth. Overexpressing SMAD3 in SKBR3 cells reduced the sensitivity of tumor cells to T-DM1 treatment (Fig. 8F, G).

To further validate these targets, first, we generated reporter constructs for SMAD3 and cotransfected them with the corresponding lncRNAs and assessed the SMAD-responsive transcriptional regulation of LINP1, BREA2, and EGOT via a luciferase reporter assay. The SBE reporter indicated that SMAD3 overexpression resulted in a significant increase in SMAD activity in T-DM1-treated cells, whereas ASO-only treatment abrogated the decrease in luciferase activity (Fig. 8H–J and Supplementary Fig. S5). Finally, we validated the FOXO1 target by generating a reporter construct, and luciferase experiments also indicated that FOXO1 luciferase activity increased in T-DM1-treated tumor cells (Fig. 8K and Supplementary Fig. S5). Together, these data clearly indicate that SMAD3 and FOXO1 are potential target genes for LINP1, BREA2, EGOT, and LINC01503, respectively.

## Discussion

Currently, there are limited treatment alternatives available in the clinic for patients with HER2+ mBC who develop resistance to T-DM1. Given that HER2 is the only marker available for anti-HER2-based treatment in clinical practice, investigating the biological basis of T-DM1 resistance and identifying a novel target for preventing T-DM1 resistance are indispensable. Additionally, separating patients who could benefit from T-DM1 treatment and identifying non-responders for alternate courses of treatment are urgently needed. In this study, with the use of patient blood serum, we were able to identify a treatment outcome-associated signature based on 4 lncRNAs in HER2+ mBC patients who were receiving T-DM1 treatment. The 4-lncRNA signature effectively differentiated low and high ORRs, patient time to PFS, and OS. Our findings demonstrate that this proposed classifier tool can effectively segregate patients and predict the therapeutic efficacy of T-DM1 in patients with HER2+ mBC.

The specific causes of T-DM1 treatment failure in patients with HER2+ mBC remain unclear, and clinicopathological factors show relatively poor performance in distinguishing patients who are at high risk of developing resistance to T-DM1. Several studies have documented the diverse functions of lncRNAs in regulating drug resistance. Each lncRNA has specific roles in resistance mechanisms, and these findings raise the possibility that lncRNAs could serve as potential prognostic biomarkers for breast cancer patients [21–23]. Despite the fact that many lncRNA biomarker studies have been reported using data from public databases, none of these studies were subjected to large-scale, high-throughput clinical samples and independent validation. Importantly, no studies have investigated the role of serum lncRNAs in predicting the T-DM1 response in patients with HER2+ mBC. In the present study, we adopted a three-step strategy to develop and validate an lncRNA signature as a predictive biomarker for the T-DM1 response in HER2+ mBC patients. First, a microarray was used to identify differentially expressed lncRNAs in the T-DM1-sensitive and T-DM1-resistant groups. Resistance-related lncRNAs were discovered in the training cohort (discovery cohort). All patient serum samples in the training cohort were subjected to qRT-PCR to quantify the selected lncRNAs in a larger cohort, and a 4-lncRNA signature panel was developed. Finally, we validated the performance of the 4-lncRNA signature in two independent validation cohorts (the internal cohort and independent cohort). To compare treatment outcomes, a separate chemotherapy-only cohort was used. Considering all the evidence presented here, we suggest that the proposed lncRNA signature is a promising tool for predicting T-DM1 resistance in HER2+ mBC patients.

Ideally, a robust prognostic biomarker of treatment outcomes should, in theory, be able to identify a subset of patients early enough in the course of treatment to warrant consideration of alternative treatment and who are at high risk of both disease relapse and treatment resistance. Additionally, T-DM1 treatment might have unrealized clinical benefits for HER2+ mBC patients who are masked owing to a lack of biomarkers in the selection of T-DM1-responsive patients. Identifying reliable predictive and prognostic biomarkers is therefore desperately needed. The evidence we provide here indicates that the 4-lncRNA signature exhibited 74% specificity and 73.8% sensitivity in predicting the therapeutic effects of T-DM1. Notably, a 4-lncRNA signature plays a dual role in prognostic and predictive measures for patients with HER2+ mBC; patients with high-risk scores are more likely to experience recurrence and will benefit most from T-DM1 therapy.

LncRNAs are involved in multilevel regulation of gene expression and have evolved as numerous hallmarks of cancer metastasis [10, 24] and [25]. Nevertheless, the functions of lncRNAs in T-DM1 resistance remain poorly understood. Conversely, the roles of the 4 lncRNAs in our signature are largely undefined, except for LINP1, which has been reported to regulate DNA double-strand break [26] and tamoxifen [27]. Our integrated analysis revealed a key function that is dysregulated in HER2+ mBC patients. This study further provides an overview of the functional enrichment of lncRNAs that are abundantly expressed in treatment-resistant and treatment-sensitive contexts and that target genes that are involved in RNA processing, TGF-beta signaling, inflammatory pathways, and RNA transport. Recent studies have reported that infiltrating lymphocytes and immune gene signatures in primary tumors predict the benefit of trastuzumab in patients with HER2+ early [28] and advanced breast cancer [15, 29]. This finding led us to examine the variations in immune infiltration levels between the resistant (high-risk) and sensitive (low-risk) groups via the lncRNA signature. Our findings revealed that the infiltration of resistant CD8 + T cells and B cells was inadequate. Second, we analyzed the origins of these immune cells by assessing exosome and exosome-free supernatants derived from primary breast cancer cells and peripheral immune cells. Interestingly, our findings indicate that the lncRNAs LINP1 and EGOT are released from tumor cells, whereas BREA2 and LINC01503 primarily originate from the exosomes of immune cells. These findings suggest that our lncRNA signature, which may be attributed to the immunological association and origin of these lncRNAs, may effectively distinguish between resistant (high-risk) and sensitive (low-risk) groups. Furthermore, our findings imply that these four lncRNA signatures are functional regulators of T-DM1 treatment outcome in HER2+ mBC cells in addition to being predictive biomarkers.

The 4-lncRNA signature derived from serum performed better than the single lncRNA signature and other clinical parameters, such as age, metastasis status, menopausal status, Ki-67 rate, estrogen status, and progesterone receptor status. While these parameters reflect anatomical differences, we speculate that lncRNAs may reflect the biological heterogeneity and immunological diversity of tumors, offering deeper insight into patients’ systemic conditions. Taken together, our findings highlight the importance of immune cell infiltration in T-DM1 efficacy as well as the potential functions of circulating immune cell exosomes in mediating T-DM1 resistance.

Our study offers several benefits. First, we detected lncRNAs in serum samples via microarray analysis. This technique is more affordable than RNA sequencing, fast, technically reliable, widely applied in hospital clinical settings, and constantly yields novel lncRNA discoveries. Second, the advantage of microarrays is that a modest amount of RNA is needed as input material. Importantly, serum lncRNAs are less abundant than they are in tumor tissue samples with respect to prognostic identification. Third, we have developed lncRNA signatures on qRT-PCR data that are highly feasible in clinical settings and may be used in the majority of hospitals. Last, multicenter validation is applied to this investigation, providing the usefulness and effectiveness of this promising tool.

## Conclusions

In conclusion, our findings suggest that the serum-based 4-lncRNA prognostic tool can accurately distinguish HER2+ mBC patients who are either sensitive or resistant to T-DM1, outperforming other clinical indicators. The signature is fine-tuned via microarray expression data from patient serum, quantified via RT-PCR, and could be a useful tool for clinical application as a biomarker for predicting T-DM1 efficacy.

## Methods

### Patient enrollment and study design

This study was approved by the Institutional Ethics Review Boards of KFSH&RC (RAC # 2210002) and Park View Hospital Chattogram, Bangladesh (BMRC/ERB/Approval/2026-2025/93). Before blood samples were collected, each patient provided signed informed consent. We collected blood serum samples from 250 HER2+ metastatic (mBC) patients and 50 healthy controls. The following eligibility criteria were strictly followed. (1) Metastatic disease with pathologically confirmed adenocarcinoma of the breast. (2) The patient’s immunostaining HER2 positivity score was 3(+). (3) The amplification of fluorescence in situ hybridization (FISH, HER2/CEP17) ratio was greater than 2. (4) The patient received T-DM1 as first-line therapy. (5) Follow-up time or life expectancy longer than 6 months. (6) Eastern Cooperative Oncology Cohort (ECOG) scale score less than 2.

The serum samples were obtained before T-DM1 treatment from patients treated with KFSH&RC (85 samples total) and allocated to the training cohort. An additional 165 samples from Park View Hospital, Chattogram, Bangladesh, were randomly classified into the external validation cohort. In addition, paraffin-embedded tumor samples were obtained from 110 patients at KFSH&RC and Parkview Hospital. Additionally, we collected 100 serum samples from patients who received only chemotherapy. All patients received T-DM1 at a concentration of 4.5 mg/kg^−^^1^ loading dose and 2 mg/kg^−^^1^ weekly or 8 mg/kg^−^^1^ loading doses and then 6 mg/kg^−^^1^ every 3 weeks. ASCO recommendations were followed for the administration of chemotherapy regimens. The response rate (RR) was evaluated via the Response Evaluation Criteria in Solid Tumors Criteria version 1.1 [30]. When the disease progressed in less than 6 months, it was considered treatment resistant. On the other hand, treatment sensitivity was defined as a complete response (CR), partial response (PR), or stable disease (SD) that was prolonged for more than 6 months over the follow-up time. The criteria utilized in several earlier clinical trials, either with or without T-DM1 treatment, were the basis for the aforementioned definition [1, 29, 31].

### Blood/serum sample collection and processing

The early detection research network serum standard operating protocol was followed for the collection and processing of the serum samples. (1) Blood samples were collected from all patients while they were fasting, placed into a serum separator tube, labeled, and gently inverted 5–7 times. (2) Allow the blood samples to clot spontaneously at room temperature for 15 to 20 min. (3) Centrifuge the tube at 1000 × *g* for 15 min in a refrigerated centrifuge. (4) The separated serum was transferred to an RNase-free tube, and (5) the serum was frozen at −80 °C until use.

### RNA extraction, microarray, and bioinformatics analysis

Total RNA from each serum sample was extracted via TRIzol (Invitrogen, USA), the RNA concentration was quantified via a NanoDrop ND-100, and RNA integrity was assessed via standard agarose gel electrophoresis. The Agilent platform was used for lncRNA microarray data capture. Sample preparation and microarray hybridization were performed according to the manufacturer’s standard protocol with little to no protocol modifications. Each sample was amplified and transcribed into fluorescent cRNA along the entire length of the transcripts without 3’ bias via random priming methods (Arraystar Flash RNA Labeling Kit, Arraystar, USA). Labeled cRNAs were hybridized onto the Human LncRNA Array v5.0 (8x60K, Arraystar, USA). Following washing, the arrays were scanned via the Agilent Scanner G2505C. Agilent Feature Extraction software (v 11.0.1.1) was used to analyze the acquired array images. Quantile normalization and subsequent data processing were performed via the GeneSpring GXv12.1 software package (Agilent Technologies, USA). Following transcript normalization, the lncRNA-specific probes were flagged as “present” in more than or equal to half of the samples and were considered highly qualified for biomarker selection. Differential expression was determined via a *t*-test between samples from 10 resistant patients and 10 sensitive patients. Differentially expressed lncRNAs between the two groups were identified with cutoffs of *p* < 0.05 and a fold change >1.5.

### qPCR analysis

Total RNA was extracted from 500 μl of serum from each sample via a plasma/serum circulating and exosomal RNA Purification Mini Kit (42800, NorgenBiotek Corporation, ON, Canada) following the manufacturer’s instructions. Total RNA was reverse-transcribed via an iScript cDNA synthesis kit (1708891, Bio-Rad, USA). The complementary DNA was reverse-transcribed via a cDNA reverse transcription kit (Thermo Fisher Scientific, USA) as previously described [32]. The cDNA was analyzed by qPCR using Invitrogen Platinum SYBR Green SuperMix (Thermo Fisher, USA) with a QuantStudio 3 Applied BioSystem Real-Time PCR system (Thermo Fisher, USA). After initial denaturation at 95 °C for 5 min, 45 cycles of the following program were used for PCR amplification: 95 °C for 15 s, 60 °C for 15 s, and 95 °C for 15 s. After the qPCR run was completed, the raw qPCR data were obtained and analyzed with Design and Analysis (DA2) software. LncRNA expression was measured via the delta‒Ct method, with GAPDH serving as the internal control gene. The list of PCR primers used in this study is listed in Supplementary Table 1.

### Development of a serum lncRNA-based signature for T-DM1 treatment response

The data from the training (discovery) cohort were used to create the serum lncRNA-based treatment prediction model, which was then applied to both the internal (test) and independent (validation) cohorts. First, we employed univariate Cox analysis to identify serum lncRNAs associated with treatment outcomes to develop a serum-based signature. For regression with high-dimensional predictors, least absolute shrinkage and selection operator (LASSO) is a popular alternative method. Coefficient estimates can be forced to approach zero by using the L1 penalty, thereby reducing the number of candidates [33, 34]. To further reduce the number of candidates, this strategy has been embraced and widely used with the Cox proportional hazard regression model for survival analysis and for optimal selection of candidate genes in a high-dimensional dataset [35, 36]. To achieve shrinkage and variable selection simultaneously, we adopted a Cox regression model with the LASSO penalty method. Repeated tenfold cross-validation was used to determine the optimal *λ* value [35–37]. On the basis of the *λ* value, we selected the lncRNAs whose beta coefficients were not zero to calculate the risk score for each patient. The “glmnet” package from R software version 4.2.0 (R Foundation for Statistical Computing) was used to perform LASSO regression analysis. The risk score was generated via a formula derived from the expression levels of these lncRNAs weighted by their coefficients, and then a time-dependent receiver operating characteristic (ROC) curve [37, 38] was used to determine the predictive accuracy via the “pROC” package [39] in R software. Patients were categorized into low- and high-risk groups with the threshold that produced the maximum sum of sensitivity and specificity in the ROC curve.

### X-Tile analysis

The X-tile plot provides a single and intuitive method to assess the associations between variables and survival. Using X-tile software version 3.6.1 (Yale University School of Medicine, New Haven, CT, USA), we selected the optimal cutoff score for the expression of each lncRNA on the basis of its association with patient disease-free survival and overall survival. The X-tile program can automatically select the optimum data cutoff point according to the highest *λ*^2^ value (minimum *p* value) defined by Kaplan‒Meier survival analysis and the log-rank test [40].

### Functional enrichment analysis

The functional context of the serum lncRNA signature was explored on the basis of the guilt-by-association principle [37, 41]. This approach is based on correlation analysis between matching lncRNA and mRNA expression in combination with enrichment strategies. It includes three steps. (1) For each lncRNA, an expression-correlation matrix (Spearman’s rank) was constructed by integrating the lncRNA and matching mRNA expression data. (2) The expression-correlation matrix was ranked by the correlation coefficient in descending order, and (3) the sorted expression-correlation matrix was used as input for GSEA, a hallmark gene set list obtained from MSigDb and immune-related pathways from the ImmPort website [14] with the “clusterProfiler” package [42] in R software. Pathways with *p* value < 0.05, false discovery rates (*q*-value) < 0.25, and absolute normalized enrichment scores (NESs) > 1 were considered significant and were used to infer the functions of the corresponding lncRNAs.

### Integrative lncRNA:mRNA expression analysis

Integrative lncRNA:mRNA analysis was carried out as previously described [43, 44]. We took into account samples for which we had both lncRNA and mRNA microarray data. Briefly, a Spearman correlation coefficient (rho) score and a *p* value were generated for comparisons of expression profiles between every possible pair of lncRNAs and mRNAs. KEGG pathway enrichment of target genes of lncRNAs was performed via Fisher’s exact test. The groups of lncRNAs we considered were as follows: (1) lncRNAs that were poorly expressed in T-DM1-resistant vs. T-DM1-sensitive samples. (2) lncRNAs that were highly expressed in T-DM1-resistant vs. T-DM1-sensitive samples. Significantly enriched pathways were those with BH multiple test adjusted *p* values (*q* values) <0.05.

### Patient primary tumor cell and immune cell isolation

Primary tumor cells were extracted from freshly resected samples, and autologous T lymphocytes, B lymphocytes, NK cells, monocytes, and granulocytes were obtained from peripheral blood samples of ten (*n* = 10) HER2+ primary breast cancer patients, as previously described [15, 45, 46]. The following protocol was followed to obtain immune cells. Briefly, whole blood was diluted with phosphate-buffered saline (PBS) and then centrifuged at 400 × *g* for 15 min in Ficoll. After centrifugation, peripheral blood mononuclear cells (PBMCs) were collected from the cell layer in the interphase above Ficoll for lymphocyte and monocyte isolation, and the mixed granulocyte and erythrocyte suspension was subjected to Ficoll. Monocytes, T lymphocytes, B lymphocytes, and NK cells were purified from the PBMC fraction via CD14 microbeads (130--050--201, Miltenyi), CD3 microbeads (130--050--101, Miltenyi), CD19 microbeads (130--050--301, Miltenyi), and CD56 microbeads (130--050--401, Miltenyi), respectively, following the manufacturers’ instructions. Erythrocytes in the mixtures were lysed with ammonium chloride buffer for granulocyte isolation. Freshly resected HER2+ breast cancer cells were isolated, and the immune cells were cultured at a density of 1 × 10^6^ cells/ml.

### Cell culture, ASO transfection, and cell proliferation assay

BT474 and SKBR3 cells, two HER2+ cell lines, were obtained from American Type Cell Culture (ATCC) and used to investigate the contribution of the lncRNA signature to T-DM1 sensitivity. The cells were cultured in RPMI 1640 with 10% fetal bovine serum (FBS). The cells were transfected with lncRNA ASOs (Qiagen, USA) without the use of a transfection reagent in vitro as previously described. The ASO sequences used were as follows: LINP1-5’-CGGCACGTAGAGGACA-3’, LINC01503-5’-AGAGCTTTGGAAATGC-3’, EGOT-5’-CTGCGCTAGGTGGTAT-3’, and BREA2-5’-TCAGGCAAGAGGTGTG-3’. The sequence of the negative control ASO was 5’-ACGUGACACGUUCGGGAATT-3’. T-DM1 was obtained from the hospital clinic/pharmacy, dissolved in sterile water, and added to culture medium (8 μg/ml) for 2 days, and cell proliferation was assessed via a WST-1 assay kit.

### Invasion and colony formation assays

Invasion and colony formation assays were performed as previously described [47]. Cultured BT474 and SKBR3 cells were treated with lncASO, ASO-control, or T-DM1 (4.00 μg/ml) for 48 h. Harvested cells were seeded into the upper chamber of Matrigel-coated chambers at a density of 50,000 cells/well. After 24 h of incubation, the cells on the upper inserts were carefully removed via cotton swabs. Invaded cells were stained with 1% crystal violet for 30 min at room temperature and then washed with PBS. Invaded cells were counted under a phase contrast microscope and quantified via NIH ImageJ. The experiments were performed in triplicate. The clonogenic assay was performed on BT474 and SKBR3 cells treated as described above. The cells were trypsinized and resuspended in medium containing 40% methylcellulose (Stem Cell Technologies) supplemented with RPMI-1640 and 10% FBS. The cells were then plated in tissue culture dishes and incubated for 2 weeks. After 2 weeks, the number of colonies was counted via ImageJ.

### WST-1 cytotoxicity and proliferation assay

WST-1-based cytotoxicity and proliferation assays were conducted as previously described [48]. Briefly, cells were seeded in a 96-well plate and treated with T-DM1 and the corresponding ASOs. Cell viability was assessed with WST-1 reagents following the manufacturer’s instructions (Invitrogen, USA). The absorbance (450 nm) was measured after 48 h. Dose-response curves (DRCs) and the GRmetrics package were used to generate DRC curves via R statistical software (version 4.4.1).

### LncRNA target prediction, siRNA transfection, overexpression vector, and luciferase assay

Smad3 siRNA and FOXO1 siRNA were obtained from Origene (Maryland, USA). Lentivirus-based vectors containing SMAD3 and FOXO1 ORFs and corresponding controls were used for transfection. Both siRNAs and plasmid vectors were transfected via Lipofectamine 2000 (cat. # 11668019; Thermo Fisher Scientific) according to the manufacturer’s instructions. Twenty-four hours after transfection, the cells were lysed, and luciferase activity was measured via a dual-luciferase assay kit (Promega). The SBE reporter (TGF-beta/SMAD signaling pathway) (BPS Bioscience) was transfected separately, and luciferase activity was measured at 24 h after transfection.

### Isolation and characterization of exosomes

Primary breast tumor cells and immune cells were cultured in culture medium for 48 h, and the resulting exosomes were purified via differential ultracentrifugation from the culture supernatants via centrifugation at 2 × 10^3^ × *g* for 30 min at 4 °C to remove the cells and again spun at 1 × 10^4^ × *g* for 30 min at 4 °C to remove cellular debris. The resulting supernatants were filtered through 0.2 μm filters (Millipore, USA), followed by ultracentrifugation at 1 × 10^5^ × *g* for 60 min at 4 °C. The pellets were resuspended in PBS. Exosomal RNA extraction and qRT-PCR were performed as described above.

### Statistical analysis

We applied a two-tailed Student’s *t* test and significance analysis of the microarray data to analyze the differential expression of the lncRNAs detected by the microarray. The *χ*² test or Fisher’s exact test was used to compare the categorical variables and contingency tables. We applied univariate Cox regression analysis to distinguish clinical characteristics associated with clinical prognosis, and multivariate Cox regression analysis was applied to detect independent prognostic factors. We used the “glmnet” package to perform the LASSO Cox regression model to select the optimal weighting coefficient via penalized maximum likelihood and build a prognostic signature. The primary endpoint of this study was PFS, and the secondary endpoint was OS. PFS was defined as the period from the first date of treatment to the first relapse of breast cancer at any site, death from breast cancer, or treatment. OS was defined as the period from the first date of treatment to the date of death from any cause. The Kaplan‒Meier method and log-rank test were used to estimate and detect the probable prognostic performance among different groups, and Cox regression analysis was used to calculate the hazard ratio (HR). The area under the curve (AUC) was employed to demonstrate the sensitivity and specificity of different variables by risk estimation, and the area under the curve (AUC) at different cutoff times was used to analyze the predictive accuracy. ROC curves were generated to test the efficiency of our model and other factors via the “pROC” package. The bootstrap test was used to compare the significance of the AUC. X-tile plots were used to generate the optimum cutoff point for continuous variables. All the statistical tests were conducted with R software version 4.2.0, and a *p* value was set at <0.05 for statistical significance.

## Supplementary information

Supplemental material: Supplementary Figures and Figure legends

Supplemental material: Raw Western blot image

## Data Availability

The publicly available hallmark gene lists used in this study are available in the Molecular Signature Database (MSigDB) database (https://www.gsea-msigdb.org/gsea/index.jsp), and the immune-related gene lists are available on the ImmPort website (https://www.immport.org/home). The microarray data used in this study have been deposited in the Gene Expression Omnibus database under the accession code GSE274302. The remaining data are available within the article and Supplementary Information. All other data used to analyze and reach the conclusions of the study during the current study are available from the corresponding author upon reasonable request.
