# Supplementary material for: A long noncoding RNA-based serum signature predicts ado-trastuzumab emtansine (T-DM1) treatment benefit in HER2+ metastatic breast cancer patients: a multicenter cohort study
Source: Cell Death Discov. 2025 Sep 9;11:421. doi: 10.1038/s41420-025-02701-8 (PMC12420834; doi:10.1038/s41420-025-02701-8)
Supplement: Supplementary file 1 — Supplemental material: Supplementary Figures and Figure legends [file 41420_2025_2701_MOESM1_ESM.pdf]

A long noncoding RNA-based serum signature predicts ado-trastuzumab emtansine (T-DM1) treatment benefit in HER2+ metastatic breast cancer patients: a multicenter cohort study.

Syed Islam, et al.

Corresponding author: sislam83@kfshrc.edu.sa

This PDF includes
Supplementary Figures S1 to S5
Supplementary Tables S1 to S3
Original western blot image (1)

Supplementary Figures and Legends

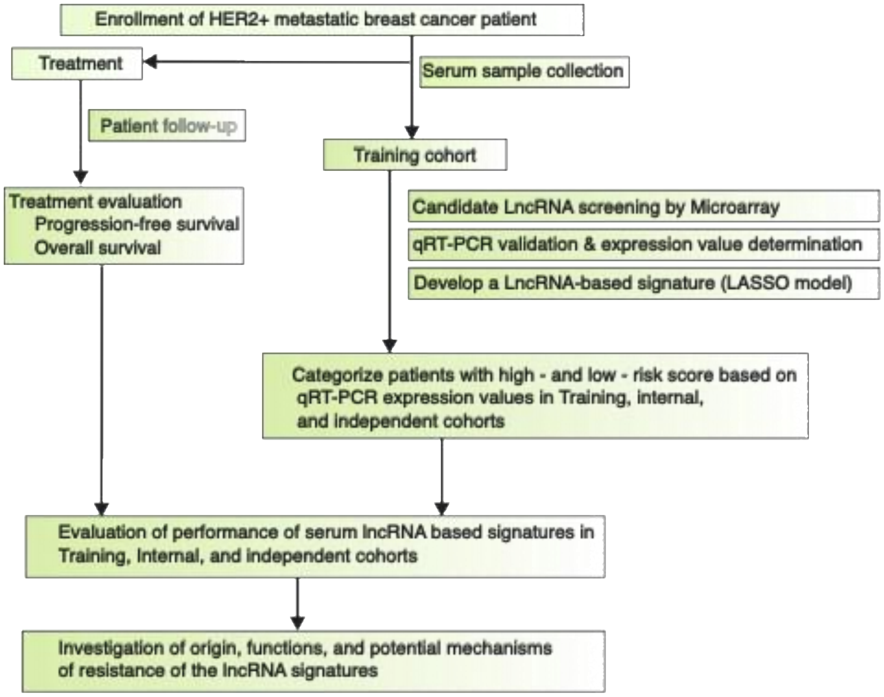

Supplementary Figure S1. Study design. The figure outlines the workflow and procedures of the REMARK diagram for building the predictive signature of the study.

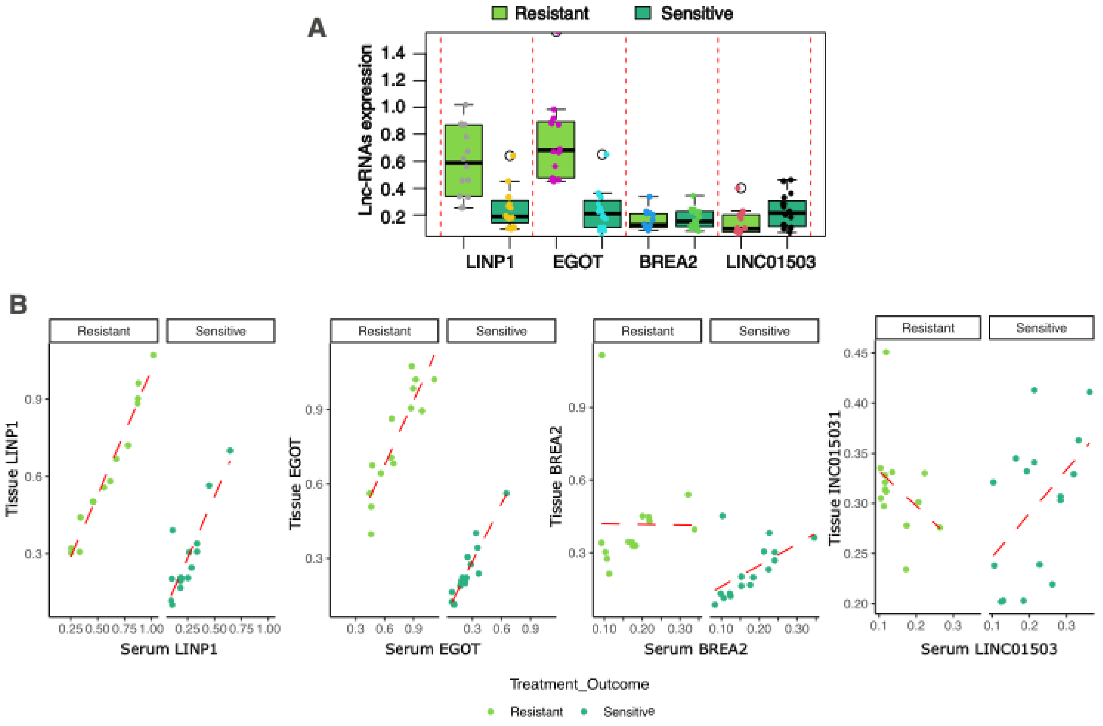

Supplementary Figure S2. Validation of the 4-lncRNA signature in tissue samples in the training cohort (n = 30). **(A)** Box plot showing the indicated tissue lncRNA expression levels in HER2+ mBC patients who received T-DM1 (resistant; n = 15; sensitive; n = 15). **(B)** The correlation between the relative expression of the indicated lncRNAs in serum and matched breast tumor tissue. Scatter plots show a positive correlation.

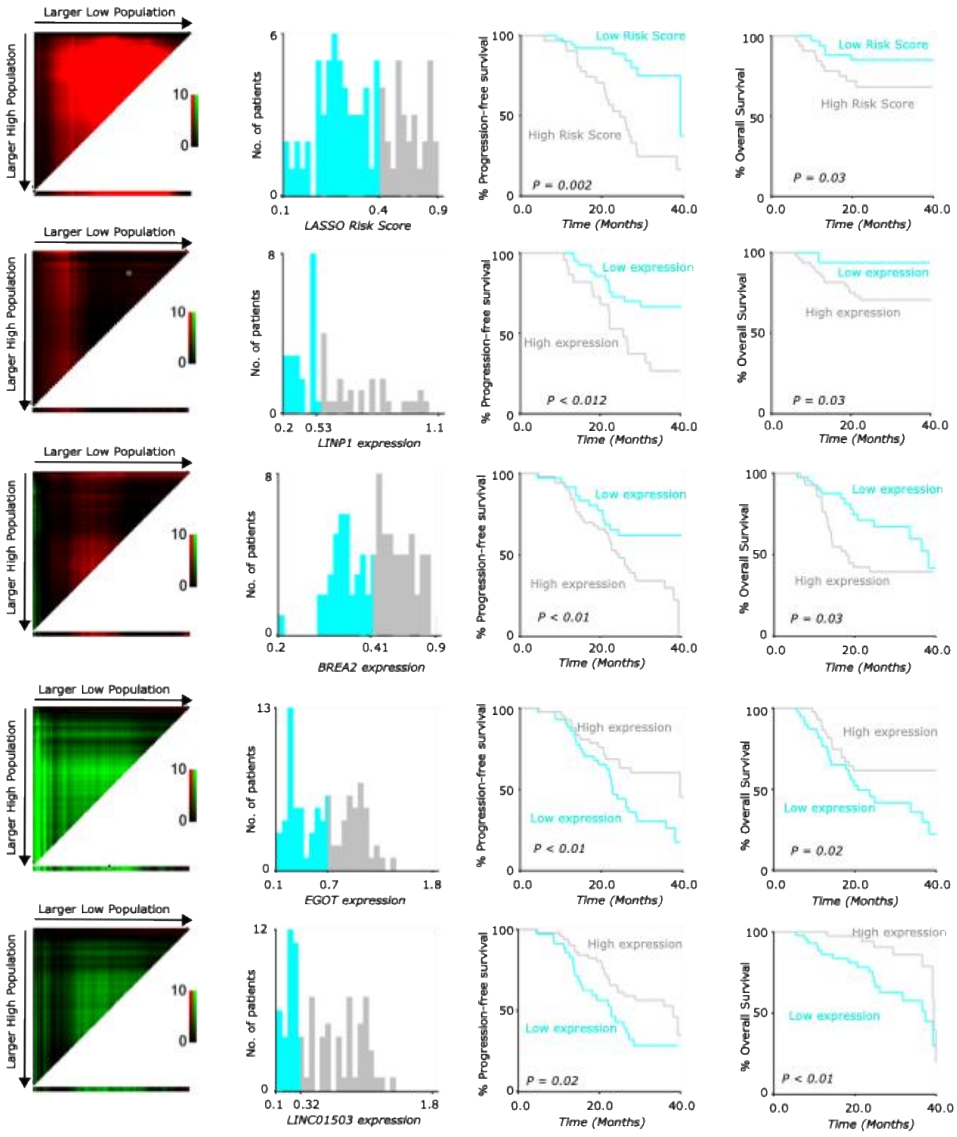

Supplementary Figure S3. The optimal values of the 4-lncRNA signature and LASSO risk score for the training set were calculated via X-tile plots. The power of the association at every division is represented by the plot colour. Green represents a direct association, and red represents a reverse association between marker expression and survival.

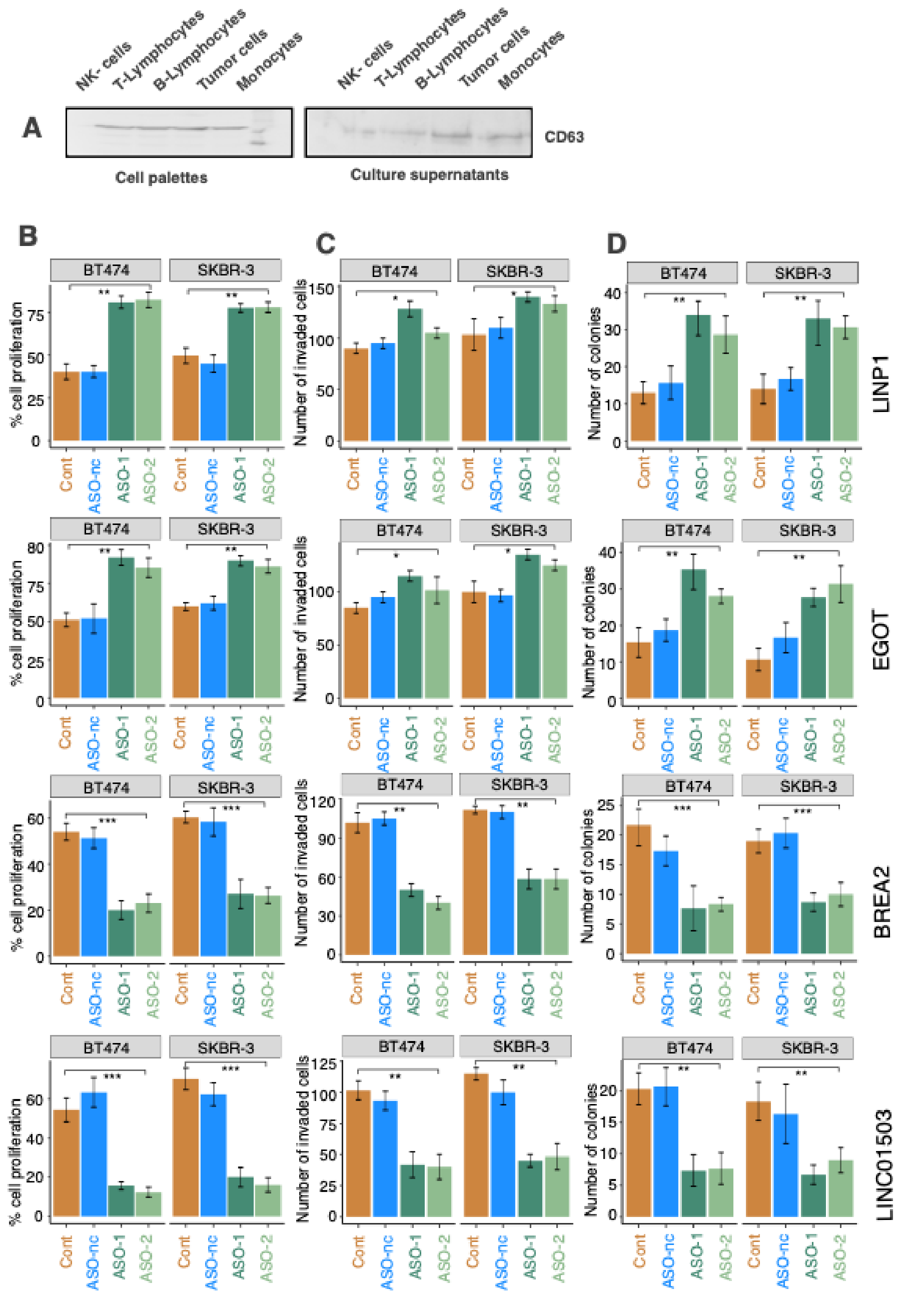

Supplementary Figure S4. (A-D) Western blot of CD63 in cell pellets and culture supernatants (A). Invasion and colony formation **(B, C)** assays of lncRNA-knockdown and ASO-nc cells. The cells were treated as described in **(D)** (mean +/- s.e.m., n = 3 separate experiments). $^*P < 0.05$, $^{**}P < 0.01$, $^{***}P < 0.001$. P values were obtained via two-tailed Student's t-test.

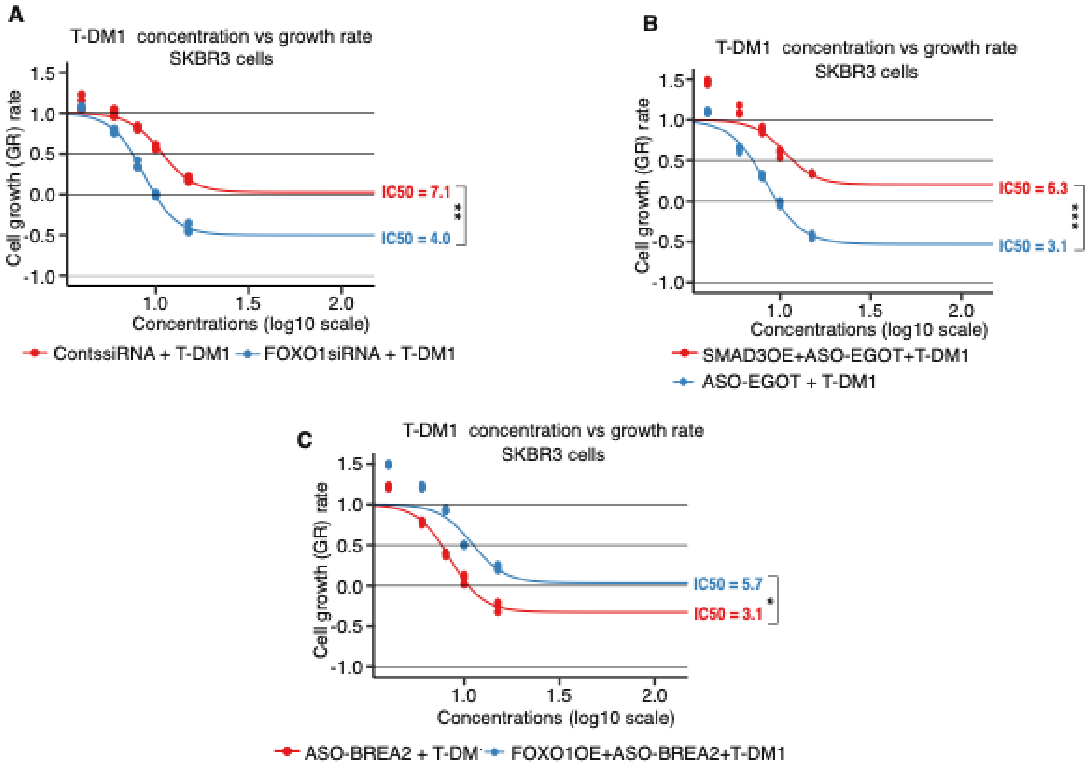

Supplementary Figure S5. (A-C) A WST-1 dose–response assay was used to assess the growth of cells in the presence or absence of ASOs, siRNAs, and vectors. (mean +/- s.e.m., n = 3 separate experiments). ***P < 0.001. P values were obtained via two-tailed Student's t-tests.

Figure 8B: Raw western blot for Aleix in primary cell pallets

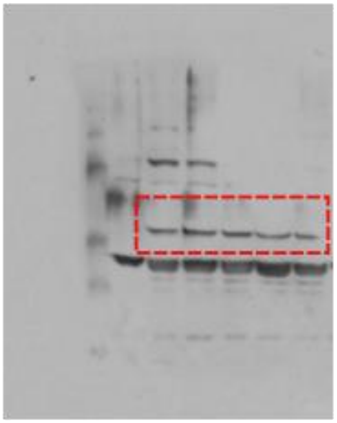

Figure 8B: Raw western blot for Aleix in culture supernatants

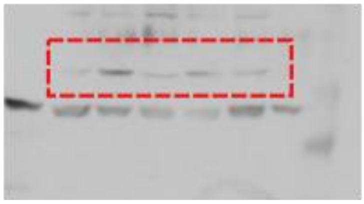

Supplementary : Raw western blot forCD63 in primary cell pallets

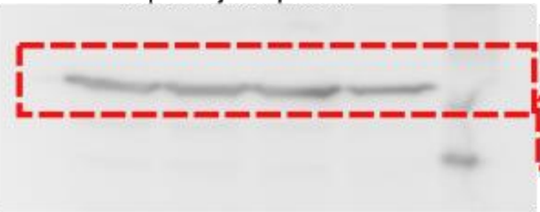

Supplementary : Raw western blot forCD63 in culture supernatants

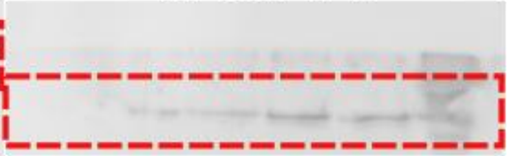

Figure: Original western blot image

Supplementary Table S1: List of forward and reverse primers.

LncRNAs	Forward primer	Reverse primer
EGOT	CCGCGCTTCAGTGGTTATGA	TGGGGAATGCAAGTTAACCGT
LINP1	AGCCGGTCAGTACACCTTT	GGAAAAGCACCGTCTGTTGTT
LINC00511	ACCCACGACCTCTTCTCCAT	GGATCAGTCCTTCCCATCCTCT
LINC01139	AGACAAGGACCCCGTCTTCA	TGGTTGGAGTTTGATGGCTGT
GDNF-AS1	CACCGAGAAGGAGAGTACTGGA	AGAACTGCACAGAAGGCTGG
MMP24OS	TGACCACCCCATCCCTTTCT	TCTCCATGACCTGGGACATGA
TINCR	AGCCTAGATCTCACTCCAGGGTCT	TGAAGCAGTGTGCATCAGCT
DNAJC27-AS1	ACCCTTCGAGATCTCTGATGGA	AAACTGACGTCCACAGCTCC
CD27-AS1	TTACAGGACAAGGAGAGGGACA	CCTGGGAAGCCACAAGGAAA
ITFG2-AS1	GCAGCAAGTTACGTCACTGC	CTTCCAGCTCTGCATCATCAGA
FGF13-AS1	AACAACCCGGAGAACCCTCA	GCTTCCTTACAGCTCCTCTGAG
LINC01128	CTGGGACCAAAGACATCTAGGC	TCAGGGTCAGGGTTCATCAGT
PATCHD4	GATGCAGCCACTACCAACCA	ATGACTCCATCAGAGCTTCTGGCTT
HDAC6	AGTTGCAAATCTGGTGATGACCTC	GCGCACATCTTGCTGAAAGG
AC009506.1	TCCCGGGGAAGAACTGCTAA	GCCAACAGTTTTGCTTCTGGG
NRSN2-AS1	GACACCTGCCGTCAGACATT	TGTAACCCAGTAGCCCCAGT
DANCR	CGCCACTATGTAGCGGGTTT	TGCCTGTAGTTGTCAACCTGC
MYLK-AS1	AAGAGCAGGACAGCAGGTGT	TTTGGGCAGGTGACTTGAGC
GAPDH	GAAATCCCATCACCATCTTCCAGG	GAGCCCCAGCCTTCTCCATG
FGF13-AS1	AACAACCCGGAGAACCCTCA	GCTTCCTTACAGCTCCTCTGAG
AFAP1-AS1	AATGGTGGTAGGAGGGAGGA	CACACAGGGGAATGAAGAGG
LINC01128	CTGGGACCAAAGACATCTAGGC	TCAGGGTCAGGGTTCATCAGT
FOXC2-AS1	AGATGCCGTTCAAGGTTTCCTT	TGGTGGGTTGTCTCAACATCC

Supplementary Table S2. Her2+ breast cancer patient's clinical characteristics, treatment, and treatment response of four different cohorts [n=350]

Patients information	Chemotherapy only cohort [n=100]	T-DM1 (Traustuzumab emtensine-1) treatment			*P* value
		Training cohort [n=85]	Internal cohort [n=65]	Independent cohort [n=100]	
Age, Median, Years	54 [37-72]	45 [32-64]	49 [36-71]	55 [37-72]	*<0.01*
Menopause status, n, [%]					
Pre-menopausal	27 [27.0]	42 [64.6]	24 [63.2]	28 [28.0]	0.062
Post-menopausal	73 [73.0]	23 [35.4]	14 [36.8]	72 [72.0]	
Ki67 rate					
<20%	13 [13.0]	6 [9.3]	4 [10.5]	10 [10.0]	
>20%	79 [79.0]	59 [90.7]	34 [89.5]	81 [81.0]	0.109
Unidentified	8 [8.0]	0 [0.0]	0 [0.0]	9 [9.0]	
ECOG performance status, n, [%]					
0	63 [63.0]	45 [69.2]	27 [71.0]	64 [64.0]	
1	27 [27.0]	17 [26.2]	8 [21.0]	25 [25.0]	0664
2	10 [10.0]	2 [3.8]	3 [8.0]	11 [11.0]	
Stage, n, [%]					
I	18 [18.0]	14 [21.5]	9 [23.7]	23 [23.0]	
II	15 [15.0]	23 [35.4]	19 [50.0]	48 [48.0]	0.071
III	46 [46.0]	20 [30.8]	7 [18.4]	19 [19.0]	
IV	21 [21.0]	8 [[12.3]	3 [8.0]	10 [10.0]	
ER-status, n, [%]					
Positive	44 [44.0]	41 [63.0]	20 [52.6]	57 [57.0]	0.147
Negative	56 [56.0]	24 [37.0]	18 [47.4]	43 [43.0]	
PR-status, n, [%]					
Positive	46 [46.0]	41 [63.0]	17 [44.74]	51 [51.0]	0.153
Negative	54 [54.0]	24 [37.0]	21 [55.26]	49 [49.0]	
Number of metastatic sites, n, [%]					
1	48 [48.0]	17 [26.2]	20 [52.6]	51 [51.0]	
2	31 [31.0]	31 [47.6]	12 [31.5]	28 [28.0]	*0.018*
3 or more	21 [21.0]	17 [26.2]	6 [15.7]	21 [21.0]	
Metastatic sites, n, [%]					
Bone	16 [16.0]	12 [18.4]	13 [34.2]	21 [21.0]	
Soft tissues	42 [42.0]	21 [32.3]	8 [21.1]	34 [34.0]	
Visceral	37 [37.0]	26 [40.1]	14 [36.8]	40 [40.0]	0.343
Brain	5 [5.0]	6 [9.2]	3 [7.9]	5 [5.0]	
Chemotherapy, n, [%]					
Paclitaxel	18 [18.0]	14 [21.5]	3 [7.89]	19 [19.0]	
Docetaxel	44 [44.0]	28 [43.1]	21 [55.26]	43 [43.0]	
Doxorubicin	27 [27.0]	17 [26.2]	9 [23.68]	26 [26.0]	0.892
Capecitabine	11 [11.0]	6 [9.2]	5 [13.16]	12 [12.0]	
Response to treatment, n, [%]					
Complete response	5 [5.0]	3 [3.5]	6 [9.2]	4 [4.0]	
Partial response	16 [16.0]	24 [28.2]	17 [26.1]	28 [28.0]	
Stable disease > 6 months	30 [30.0]	9 [10.5]	12 [18.5]	18 [18.0]	
Stable disease < 6 months	13 [13.0]	12 [14.1]	6 [9.2]	11 [11.0]	*0.035*
Progressive disease	36 [36.0]	37 [43.5]	24 [36.9]	39 [39.0]	

Abbreviations: ECOG, Eastern Cooperative Oncology Cohort; ER, Estrogen receptor; PR, Progesterone receptor.

**Supplementary Table S3: Univariate survival analysis of clinical features and LncRNA signatures with OS and PFS in training set, internal set, and independent set**						
	**Training set**		**Internal set**		**Independent set**	
Clinical features	HR (95% CI)	*P* value	HR (95% CI)	*P* value	HR (95% CI)	*P* value
**Overall survival (OS)**						
Age (<45 *vs* >45)	0.98 (0.95, 1.02)	0.3	1.09 (1.01, 1.18)	0.021	0.98 (0.93, 1.03)	0.4
Menopause (yes vs no)	1.07 (0.57, 2.03)	0.81	1.78 (0.63, 5.01)	0.34	0.71 (0.32, 1.59)	0.4
Ki67 (<20% *vs* >20%)	0.65 (0.34, 1.23)	0.20	1.04 (0.37, 2.88)	0.93	0.89 (0.36 2.21)	0.8
ER (positive *vs* negative)	0.68 (0.36, 1.30)	0.25	0.86 (0.31, 2.37)	0.81	1.00 (0.46, 2.15)	0.9
PR (positive *vs* negative)	0.88 (0.45, 1.75)	0.73	0.56 (0.20, 1.59)	0.30	1.28 (0.60, 2.73)	0.5
Metastatic sites (>2 *vs* 1)	1.21 (0.61, 2.39)	0.03	1.63 (0.55, 4.82)	0.01	1.08 (0.52, 2.31)	0.8
LINP1 (high *vs* low)	1.88 (0.99, 3.59)	0.054	2.02 (0.75, 5.43)	0.02	1.87 (0.85, 4.09	0.012
EGOT (high *vs* low)	2.13 (0.98, 4.65)	0.052	0.88 (0.32, 2.44)	0.04	1.67 (0.66, 3.76)	0.03
LINC01503 (high *vs* low)	0.75 (0.23, 2.07)	0.020	1.02 (0.38, 2.74)	0.01	0.91 (0.71, 2.63)	0.007
BREA2 (high *vs* low)	1.07 (0.57, 2.06)	0.040	1.16 (0.42, 3.22)	0.001	0.86 (0.39, 1.92)	0.04
Four-LncRNA signature (high *vs* low)	2.71 (1.35, 5.44)	0.005	3.03 (0.97, 9.45)	0.005	2.60 (1.19, 5.68)	0.017
**Progression-free survival (PFS)**						

Age (<45 *vs* >45)	1.02 (0.99, 1.06)	0.20	0.97 (0.93, 1.02)	0.20	0.98 (0.94, 1.02)	0.3
Menopause (yes *vs* no)	1.73 (0.97, 3.09)	0.054	1.54 (1.03, 4.22)	0.60	1.09 (0.61, 2.05)	0.7
Ki67 (<20% *vs* >20%)	0.65 (0.34, 1.26)	0.20	0.89 (0.46, 1.72)	0.70	0.97 (0.50, 1.88	0.9
ER (positive *vs* negative)	1.33 (0.71, 2.49)	0.41	1.33 (0.68, 2.58)	0.40	1.21 (0.62, 2.35)	0.6
PR (positive *vs* negative)	1.08 (0.57, 2.05)	0.87	1.05 (0.55, 1.99)	0.90	1.47 (0.76, 2.84)	0.3
Metastatic sites (>2 vs 1)	1.32 (0.69, 2.52)	0.04	1.11 (0.56, 2.18)	0.01	1.14 (0.53, 20.6	0.03
LINP1 (high *vs* low)	1.87 (0.88, 3.27)	0.032	1.65 (0.86, 3.18)	0.013	1.97 (0.98, 3.95)	0.052
EGOT (high *vs* low)	1.90 (1.48, 3.66)	0.01	1.17 (0.67, 2.89)	0.004	0.78 (0.49, 1.56)	0.02
LINC01503 (high *vs* low)	0.61 (0.34, 1.08)	0.03	0.82 (0.41, 1.61)	0.001	0.82 (0.42, 1.61)	0.01
BREA2 (high *vs* low)	0.77 (0.43, 1.37)	0.04	0.84 (0.41, 1.73)	0.02	0.84 (0.41, 1.73)	0.02
Four-LncRNA signature (high vs low)	3.11 (1.52, 6.05)	0.001	3.17 (1.47, 5.83)	0.002	3.74 (1.88, 7.47)	<0.001

P-values were calculated with a two-sided log-rank test
HR hazard ratio, *CI* Confidence interval, *ER* Estrogen receptor, *PR* Progesterone receptor
