# Supplementary material for: A long noncoding RNA-based serum signature predicts ado-trastuzumab emtansine (T-DM1) treatment benefit in HER2+ metastatic breast cancer patients: a multicenter cohort study
Source: Cell Death Discov. 2025 Sep 9;11:421. doi: 10.1038/s41420-025-02701-8 (PMC12420834; doi:10.1038/s41420-025-02701-8)

Figure 8B: Raw western blot for Aleix in primary cell pallets

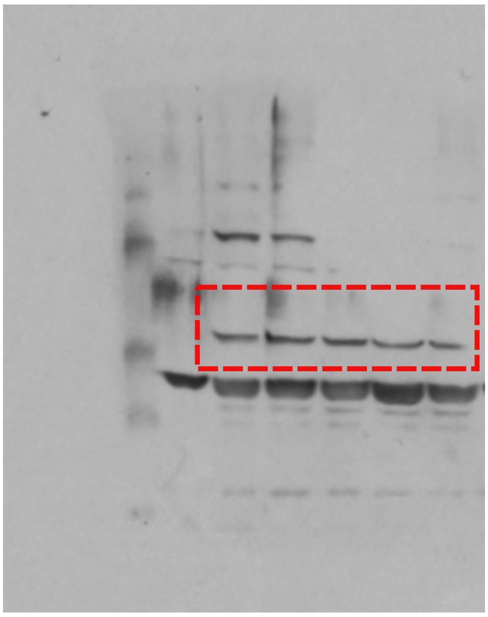

Figure 8B: Raw western blot for Aleix in culture supernatants

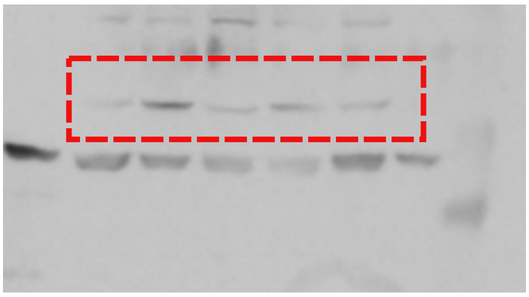

Supplementary : Raw western blot forCD63 in primary cell pallets

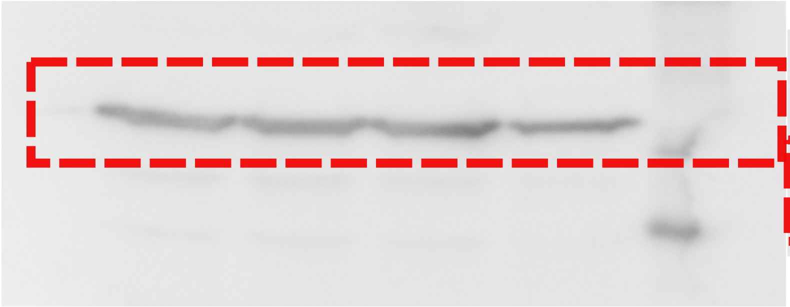

Supplementary : Raw western blot forCD63 in culture supernatants

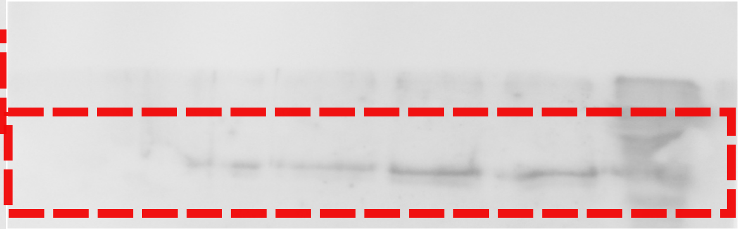

Supplement: Supplementary file 2 — Supplemental material: Raw Western blot image [file 41420_2025_2701_MOESM2_ESM.pdf]
